# Supplementary material for: Large-scale modular and uniformly thick origami-inspired adaptable and load-carrying structures
Source: Nat Commun. 2024 Mar 15;15:2353. doi: 10.1038/s41467-024-46667-0 (PMC10942996; doi:10.1038/s41467-024-46667-0)
Supplement: Supplementary file 1 — Supplementary Information [file 41467_2024_46667_MOESM1_ESM.pdf]

Supplementary Information

Large-Scale Modular and Uniformly Thick Origami-Inspired Adaptable and Load-Carrying Structures

Authors: Yi Zhu[1,2,*], Evgueni T. Filipov[2,1,*]

Affiliations:

[1] Department of Mechanical Engineering, University of Michigan, Ann Arbor, 48105, USA.

[2] Department of Civil and Environmental Engineering, University of Michigan, Ann Arbor, 48105 USA.

* Corresponding author. Email: yizhucee@umich.edu & filipov@umich.edu

Supplementary Note

S1. Summary of Different Deployable Structures

Here, we present a literature review of related deployable and reconfigurable systems used in aerospace engineering, mechanical engineering, and civil engineering. The provided material shows details regarding how we plot Fig. 1 of the manuscript. We will focus on the number of achievable shapes and packaging ratios of these systems.

Table S1. A summary of deployable aerospace structures

System	Number of Shapes	Packaging Ratio	Comments	Reference
Deployable Antenna	2	3		[1] [2]
Thick Origami Flasher	2	~5	Packaging ratio estimated with image	[3] [4]
Solar Sail	2	>100		[5]
Truss Beam	3	20	Bending configurations counted as one mode	[6]
Hoberman Sphere	2	~10	Diameter expansion ratio is around 2 to 3	[7]
Deployable Booms	2	~50		[8]

Table S2. A summary of metamorphic and reconfigurable mechanisms

System	Number of Shapes	Packaging Ratio	Comments	Ref.
8R Metamorphic Linkages	>10	~2	Limited volumetric change	[9, 10]
Spatial Linkages	>5	~10	Base Myard linkage is SDOF	[11]
Metamorphic Origami Metamaterials	10-100	NA	Not designed to have packaging or load carrying shapes	[12, 13]

First, Table S1 summarizes deployable aerospace structures using their packaging ratio and number of achievable shapes. Most of these deployable systems are unifunctional and their design is optimized for that single function. Thus, these deployable structures usually have just two configurations – a stowed shape for launching and a deployed shape for functioning. In general, thick-panel structures such as deployable antenna and solar battery tend to have lower packaging ratio while membrane-type structures can have a much larger packaging ratio. These

deployable aerospace systems are different from deployable structures for civil applications because they do not need to support the gravity load.

Next, we want to briefly discuss other metamorphic and reconfigurable mechanisms used in mechanical engineering (summarized in Table S2). MDOF linkages, such as the 8R linkage shown in [9], can achieve many shapes to build metamorphic systems. In addition, it is possible to connect SDOF over-constrained linkages, such as the Myard linkage to achieve a relatively high packing ratio and many shapes [11]. There are also metamorphic origami metamaterials that can achieve many shapes through reconfiguration [12, 13]. However, these systems were not shown to have load-carrying capability needed for civil engineering applications. Moreover, although many of these metamorphic systems can achieve a large number of shapes and configurations, there is no guarantee that the produced shapes are useful for adaptable civil structures so these configurations may not be counted as usable configurations. Compared to these metamorphic mechanisms, the proposed Modular and Uniformly Thick Origami-Inspired Structures (MUTOIS) can achieve better load carrying capability and have usable configurations to serve as civil structures.

Table S3. A summary of modular civil structures

System	Number of Shapes	Packaging Ratio	Comments	Ref.
Cargo Based Modular Structures	1	1		[14]<br>[15]
Modular Construction	1	1		[16]
Reusable Trusses	Multiple	>10	Not deployable systems (require manual assembly)	[17]

Modular construction for civil structures is another relevant research field. Table S3 summarizes different modular structures used in civil buildings and infrastructures. Cargo container modules are used to build modular civil buildings [14, 15]. These modules are built to have the size of standard cargo container for transportation. Once transported to the construction site, these cargo units are connected manually. Similar approaches are also used in the construction of steel and heavy timber structures, where individual components are shipped to the construction site and connected onsite [16, 17]. Although these systems can be seen to have a large packaging ratio (depending on how to calculate the packaging ratio), these modular structures are not deployable. The sizes of individual components within these prefabricated systems are still limited by transportation limits and they cannot change their shapes onsite. However, with the proposed MUTOIS system, it is possible to transport a packaged component and deploy it to form larger structural member onsite for functionality.

Finally, we want to summarize existing deployable and reconfigurable civil structures (see Table S4). Accordion shelter is one major form of deployable structure and has been used in

military and civil applications for more than fifty years [18]. Usually, these structures have decent packaging ratio and have two configurations: one stowed state for transportation and one deployed state for housing people. Common deployable shelters usually require 4-10 soldiers to deploy and the deployment time is between 15 minutes to a couple hours depending on the type of systems used [18]. Inflatable structures can achieve a much larger packaging ratio when compared to accordion shelters [19]. However, inflatable buildings tend to provide lower structural stiffness and strength due to the membrane constructions. In general, both accordion shelters and inflatable structures are built as enclosure structures with membrane type façade, which means that these systems are not good at supporting people or vehicles to cross on top. To resolve the load-bearing challenge, specialized deployable bridges are also built to carry large structure loads for both military operations and disaster rescue [20, 21, 22, 23]. These folding bridges have two configurations and decent packaging ratios (where the packaged length is shorter than stowed length). Actuators are used to enable rapid deployment for these bridges. Using actuators is acceptable for military applications but can be too expensive for common civil applications. Furthermore, these systems do not provide capability to reconfigure for adaptability.

Table S4. A summary of deployable civil structures

System	Number of Shapes	Packaging Ratio	Comments	Ref.
Portable Housing Shelter	2	10		[18]
Accordion Shelter	2	4	Design fits into a standard cargo container	[18]
Inflatable Buildings	2	NA		[19]
Rolling Bridge (Heatherwick)	2	~1.5	Volumetric change is small	[20]
Foldable Bridge	2	<3	Volumetric change is small	[21] [22] [23]
Deployable Shades	2	~5		[24]
Erectable Structures	2	NA	Similar to accordion structures	[25]

Figure S1 shows a summary of the packing ratio and number of configurations of the different deployable systems, including deployable aerospace systems, metamorphic mechanisms, deployable buildings, modular civil structures, military bridges, etc. This brief literature review demonstrates how we obtain Fig 1E in the main text. All references of the structures are marked on the Fig. S1.

Here, we want to briefly introduce how we calculate the packaging ratio and count the number of configurations of our MUTOIS. We will compute these numbers based on the system demonstrated in Supplementary Movie 1. Fig. S2 and S3 show how we can compute the packing ratio using the prototypes. The packed prototype shown on Fig. S2 top left is 25 cm by 56 cm by 56 cm while the deployed column configuration is 2 meter long with a cross section of around 40 cm by 40 cm. The volumetric packing ratio of this system is about 4 while the length-based

packaging ratio is around 8. In the bus stop configuration, the entire structure occupies a space that is 1 meter wide, 2 meter tall, and 2 meter long. This will bring the packing ratio to 37. In reality, we believe a packaging ratio of 6 to 8 is a more representative number of the presented system.

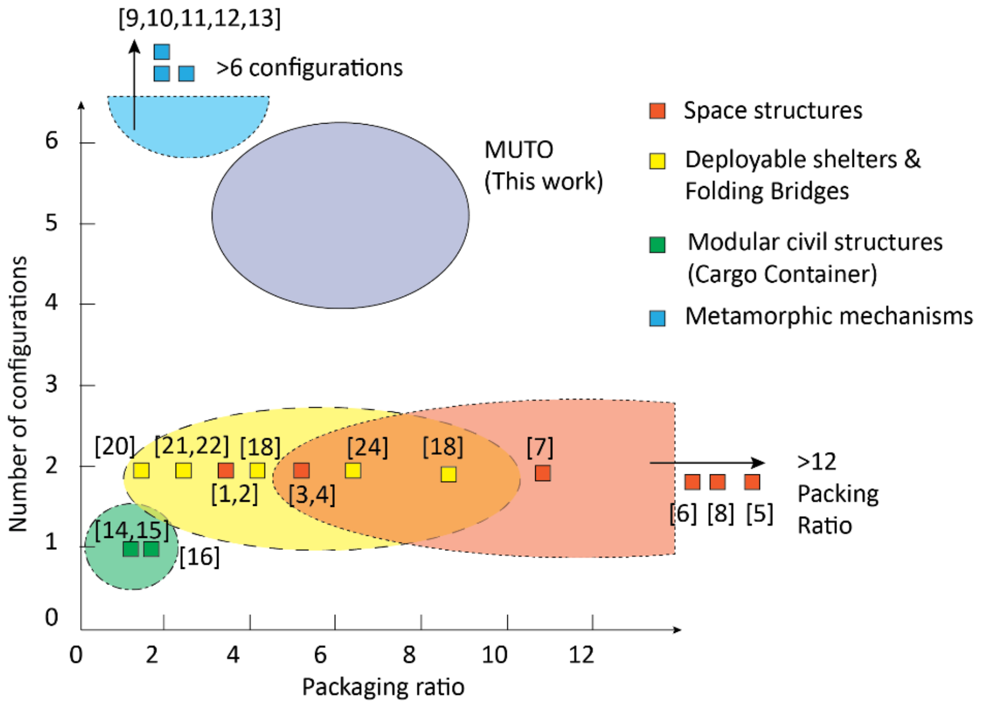

Figure S1. Summary of Packing ratio and number of configurations for deployable systems.

Next, we want to discuss the effects of panel thickness for the calculation of packing ratio. Figure S3 summarizes an idealized situation where a MUTOIS module is folded to a tubular structure. This module has a panel length of L and a panel thickness of t. The volume of the packaged configuration is $8tL^2$ while the volume of the tubular structure is $2L(L+2t)^2$. If we plot the relationship between the packaging ratio with L/t we obtain the following Fig S3. As we can see, ratio between the packaging length L and thickness t can affects the packaging ratio. Thinner panels can achieve a higher packaging ratio.

Finally, we count the number of achievable configurations of the system. The prototype shown on Supplementary Movie 1 has 7 different achievable configurations as shown in Fig S2. If we see the single column and beam configuration as one configuration and treat all bus stop shapes as one configuration, we still have 4 drastically different configurations. Technically, the full MUTOIS structure can form more shapes and configurations if we allow the system to fold along the diagonal crease lines. However, since we were not using these diagonal folds to form usable structural configurations, we do not think counting those shapes brings us a representative number. Therefore, we think 4 to 7 shapes are representative estimations for the proposed MUTOIS structures. With the provided analysis, we believe the bubble drawn on Fig 1 of the manuscript is a realistic representation of the proposed MUTOIS.

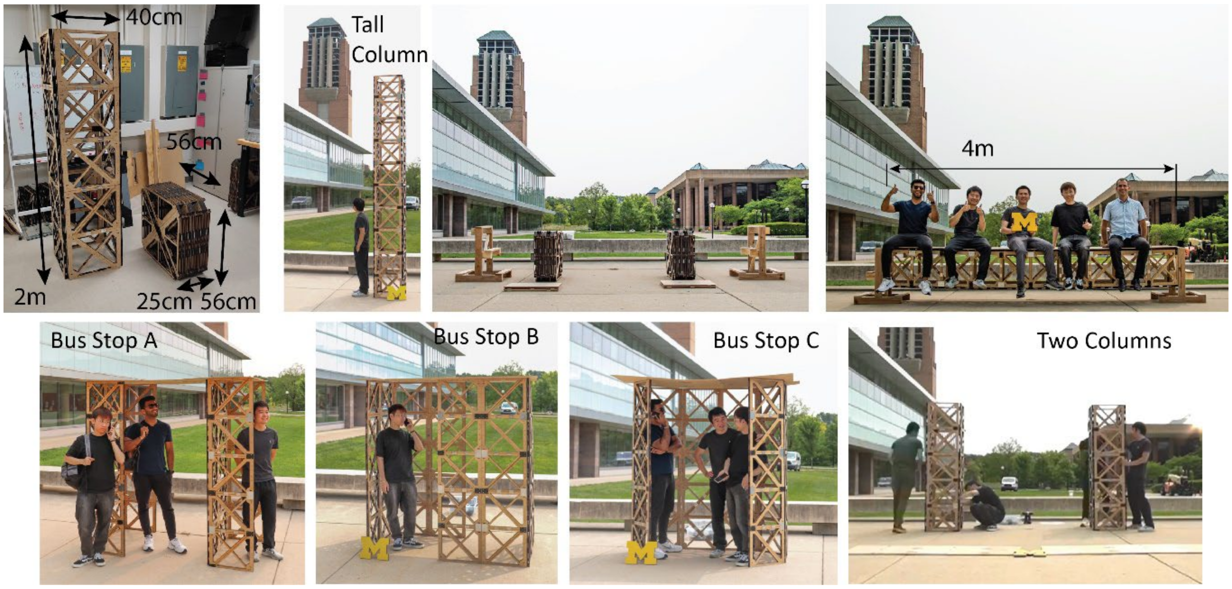

Figure S2. Calculating the number of configurations and the packaging ratio of MUTOIS.

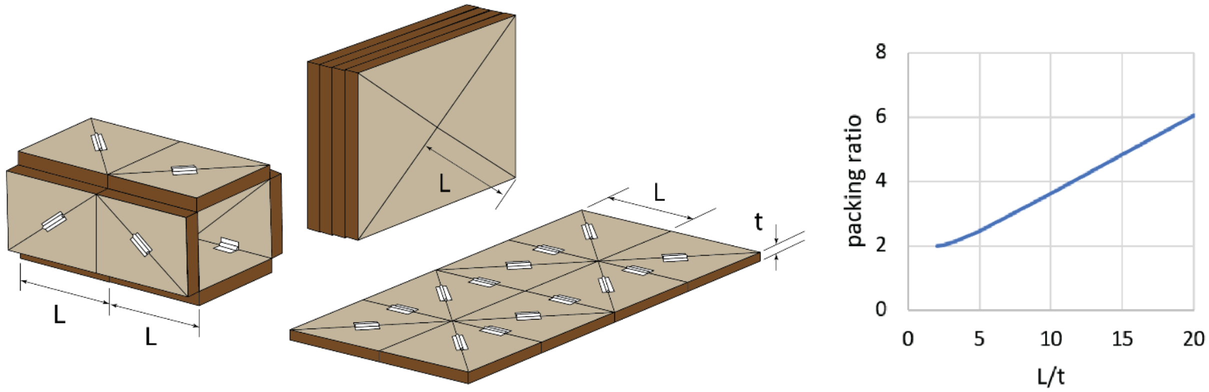

Figure S3. Calculating the number of configurations and the packaging ratio of MUTOIS.

S2. Necessary Conditions for a Thick Origami Vertex

Table S1 summarizes necessary conditions for different properties of a thick origami vertex. These conditions can ensure the thick origami vertex to have rigid foldability, single-degree-of-freedom kinematics, developability, flat-foldability, and uniform thickness. These conditions are used to design the MUTOIS. Among these conditions, this work derives Eqn. S4 and Eqn. S7, which are the necessary conditions for developability and flat foldability in thick origami vertex. We can see that the two thickness conditions share similar formulation when compared to the known sector angle conditions for developability Eqn. S3 and the Kawasaki-Justin theorem for flat-foldability Eqn. S5. Here, we will first demonstrate how we derive these two new equations and then briefly talk about other equations.

Table S5. Necessary Conditions for Thick Origami Vertex

Target Behavior	Necessary Conditions	Eqn. Number
Rigid Foldability	$\mathbf{F}(\boldsymbol{\phi}(s)) = \mathbf{T_1}(\phi_1(s))\mathbf{T_2}(\phi_2(s)) \dots \mathbf{T_N}(\phi_N(s)) - \mathbf{I_{4\times4}} = \mathbf{0_{4\times4}}$ for some $\boldsymbol{\phi}(s) \neq \mathbf{0}$	(S1)
SDOF	$\dim\left(\text{null}\left(\nabla_{\boldsymbol{\phi}}\text{flat}(\mathbf{F}(\boldsymbol{\phi}))\right)\right) = 1$ for some $\boldsymbol{\phi}(s) \neq \mathbf{0}$	(S2)
Developable	$\sum\alpha_i = 2\pi$	(S3)
	$\sum a_i = 0$	(S4)
Flat Foldable	$\alpha_1 + \alpha_3 + \dots + \alpha_{n-1} = \alpha_2 + \alpha_4 + \dots + \alpha_N = \pi$	(S5)
	$\left\lvert\sum_i C_i\right\rvert = 2$	(S6)
	$a_1 + a_3 + \dots + a_{N-1} = a_2 + a_4 + \dots + a_N$	(S7)
Uniform Thickness	$\begin{cases} a_i = t, & \text{if } C_i = -1,\ C_{i+1} = 1 \\ a_i = -t, & \text{if } C_i = 1,\ C_{i+1} = -1 \\ a_i = 0, & \text{if } C_i = C_{i+1} \end{cases}$	(S8)

In this work, we consider origami with thick panels connected by rotational hinges (see Fig. S4). These thick origami panels have two parallel surfaces (front and back of panels), which can be used to determine the normal vectors of panels. Due to the thick panel geometry – a rotational hinge is on one side of a panel – rotational hinges can only rotate towards one direction. The folding angle ϕ_i can take values between $0°$ to $180°$ for valley folds (blue hinge in Fig. S4 that fold upwards) and values between $0°$ to $-180°$ for mountain folds (red hinge in Fig. S4 that fold downwards).

To explore rigid foldability, we show the loop closure condition of a thick origami vertex:

$$\mathbf{T_1}\mathbf{T_2} \dots \mathbf{T_N} = \mathbf{I_{4\times4}}.$$

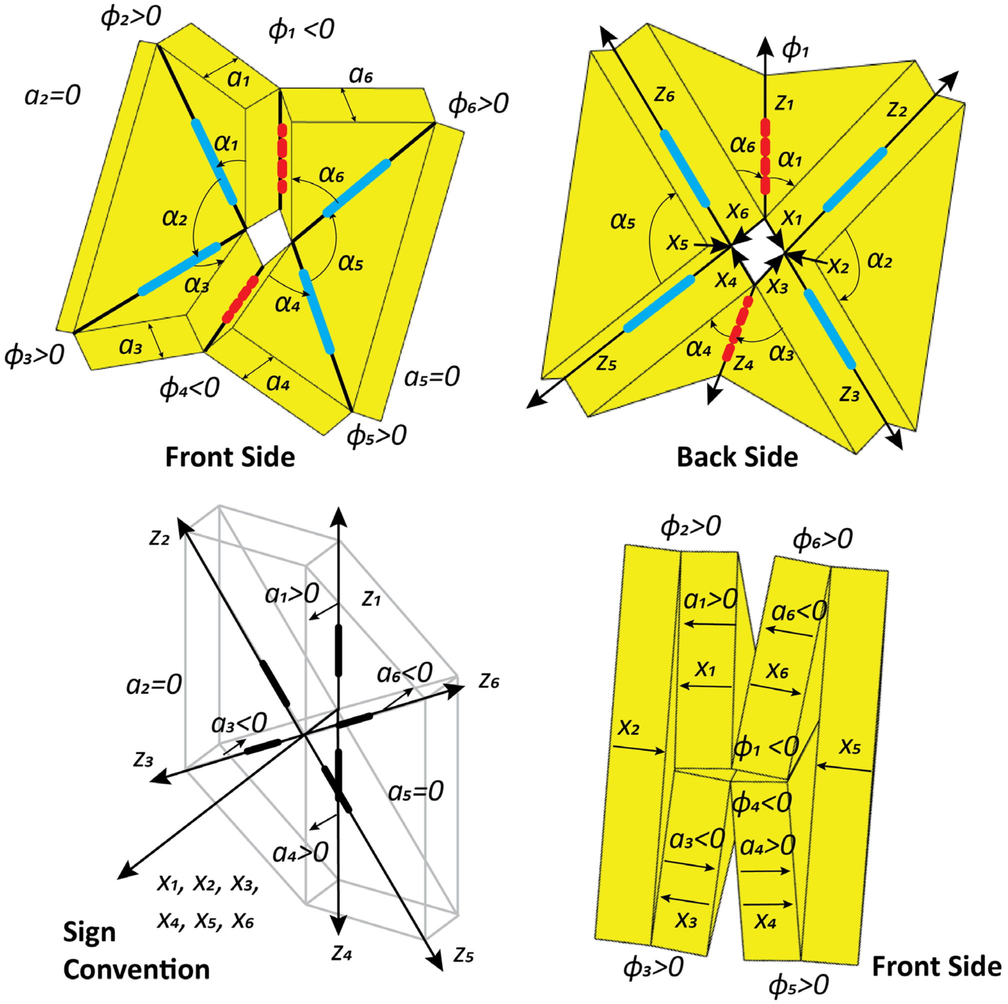

Figure S4. Notation for a thick origami vertex using the DH transformation matrix.

In this equation, the term $\mathbf{T_i}$ is the Denavit-Hartenberg transformation matrix (DH matrix), representing the rigid body motion from the rotational hinge i to the adjacent hinge $i+1$. The DH matrix can be written as follows:

$$\mathbf{T_i} = \begin{bmatrix} \cos\phi_i & -\sin\phi_i & 0 & 0 \\ \sin\phi_i & \cos\phi_i & 0 & 0 \\ 0 & 0 & 1 & r_i \\ 0 & 0 & 0 & 1 \end{bmatrix} \begin{bmatrix} 1 & 0 & 0 & a_i \\ 0 & \cos\alpha_i & \sin\alpha_i & 0 \\ 0 & \sin\alpha_i & \cos\alpha_i & 0 \\ 0 & 0 & 0 & 1 \end{bmatrix}$$

$$\mathbf{T_i} = \begin{bmatrix} \cos\phi_i & -\sin\phi_i\cos\alpha_i & \sin\phi_i\sin\alpha_i & a_i\cos\phi_i \\ \sin\phi_i & \cos\phi_i\cos\alpha_i & -\cos\phi_i\sin\alpha_i & a_i\sin\phi_i \\ 0 & \sin\alpha_i & \cos\alpha_i & r_i \\ 0 & 0 & 0 & 1 \end{bmatrix}$$

where ϕ_i is the folding angle of each crease, α_i is the sector angle of each origami panel, and a_i represents the thickness offset between adjacent creases along the **x** axis. For a common thick

origami vertex, we will have $r_i = 0$ because there is no offset along the $\mathbf{z}$ axis. Figure S4 shows the definition of each term and the sign convention with a degree-six vertex.

A different sign convention is used in this work compared to the traditional Denavit-Hartenberg convention used in [1]. With the traditional Denavit-Hartenberg convention, the x_i axis are set to have the same direction with the thickness offset a_i. Because the local coordinates need to follow a right-hand rule, the positive direction for sector angles cannot follow a uniform counterclockwise or clockwise direction – we need to set $\alpha_i^{BE} = \pi - \alpha_i$ for selected sector angles. In addition, we also need to use different equations to calculate the angle variable ϕ_i in the DH matrix from the dihedral angles, which prevents us from deriving the new thickness based necessary conditions for developability and flat-foldability. Therefore, despite being a golden practice for mechanism analysis, this traditional Denavit-Hartenberg convention is probably not the best option for our target. Here, we set the local axis following a counterclockwise direction of the sector angle α_i. With this new convention, we have a uniform representation of sector angles and fold angles so that we can derive the new constraints for developability and flat-foldability in thick origami.

S2.1 Deriving the Necessary Condition for Developable Thick Origami

We first derive the necessary thickness condition (Eqn. 3 in the manuscript) for developable thick origami. By definition, all creases are unfolded in a developed state, so we set $\phi_i = 0$ and the DH matrix for fold i becomes:

$$\mathbf{T_i} = \begin{bmatrix} 1 & 0 & 0 & a_i \\ 0 & \cos\alpha_i & -\sin\alpha_i & 0 \\ 0 & \sin\alpha_i & \cos\alpha_i & 0 \\ 0 & 0 & 0 & 1 \end{bmatrix}.$$

The thick origami should still satisfy the loop closure condition (Eqn. S1) at the flat state so we should have:

$$\mathbf{T_1 T_2 \ldots T_N} = \mathbf{I_{4\times 4}},$$

$$\begin{bmatrix} 1 & 0 & 0 & a_1 \\ 0 & \cos\alpha_1 & -\sin\alpha_1 & 0 \\ 0 & \sin\alpha_1 & \cos\alpha_1 & 0 \\ 0 & 0 & 0 & 1 \end{bmatrix} \begin{bmatrix} 1 & 0 & 0 & a_2 \\ 0 & \cos\alpha_2 & -\sin\alpha_2 & 0 \\ 0 & \sin\alpha_2 & \cos\alpha_2 & 0 \\ 0 & 0 & 0 & 1 \end{bmatrix} \ldots$$

$$\ldots \begin{bmatrix} 1 & 0 & 0 & a_N \\ 0 & \cos\alpha_N & -\sin\alpha_N & 0 \\ 0 & \sin\alpha_N & \cos\alpha_N & 0 \\ 0 & 0 & 0 & 1 \end{bmatrix} = \mathbf{I_{4\times 4}}.$$

If we extend the equation, the forth column and first row give the necessary condition:

$$a_1 + a_2 + \cdots + a_N = 0.$$

Therefore, for a thick origami to be developable, it needs to satisfy the equation above.

We want to further show that the sector angle condition for a developable thin origami vertex also applies to a developable thick origami vertex. Figure S5A explains this result. For a thin origami to be developable, the sector angles need to satisfy Eqn. S3: all sector angles should sum to 2π. If sector angles do not sum to 2π, we will have a gap or an overlap in the vertex. For the thick origami vertex, the same principle applies.

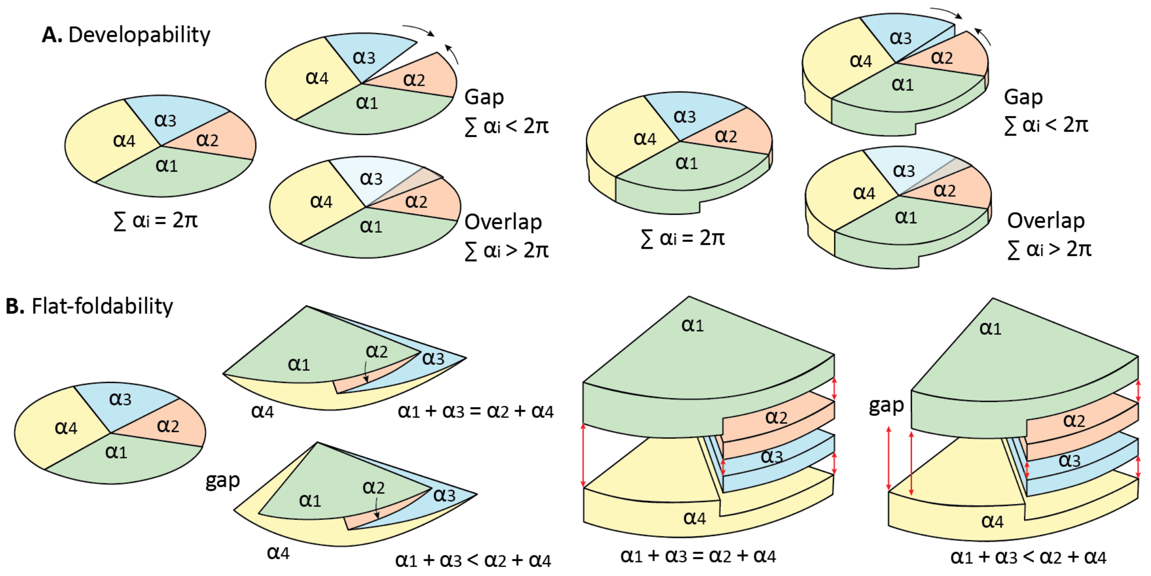

Figure S5. Sector angle condition for developable and flat foldable thin origami still apply to the thick origami vertices.

S2.2 Deriving the Necessary Condition for Flat Foldable Thick Origami

From Kawasaki-Justin theorem (Eqn. S5) and Maekawa-Justin theorem (Eqn. S6), we know that flat foldable thin origami must have an even number of creases. Moreover, the sum of odd number sector angles should equal to the sum of even number sector angles for the origami to be flat foldable (see Fig. S5B). Otherwise, a gap will form to tear the vertex. Both of these theorems still apply to thick origami structures. Although the surfaces of panels are separated due to thickness, the same analysis applies (see Fig. S5B).

Here, we further derive an additional condition for a thick origami vertex to be flat foldable. At the flat folded state, all creases within the thick origami should be folded by 180°. In this case, we will have $\phi_i = 180°$ or $\phi_i = -180°$. In both situations, we have:

$$\mathbf{T_i} = \begin{bmatrix} -1 & 0 & 0 & -a_i \\ 0 & -\cos\alpha_i & \sin\alpha_i & 0 \\ 0 & \sin\alpha_i & \cos\alpha_i & 0 \\ 0 & 0 & 0 & 1 \end{bmatrix}.$$

Then, we can put this equation into the loop closure condition (Eqn. S1) and obtain:

$$\begin{bmatrix} -1 & 0 & 0 & -a_1 \\ 0 & -\cos\alpha_1 & \sin\alpha_1 & 0 \\ 0 & \sin\alpha_1 & \cos\alpha_1 & 0 \\ 0 & 0 & 0 & 1 \end{bmatrix} \begin{bmatrix} -1 & 0 & 0 & -a_2 \\ 0 & -\cos\alpha_2 & \sin\alpha_2 & 0 \\ 0 & \sin\alpha_2 & \cos\alpha_2 & 0 \\ 0 & 0 & 0 & 1 \end{bmatrix} \cdots$$

$$\cdots \begin{bmatrix} -1 & 0 & 0 & -a_N \\ 0 & -\cos\alpha_N & \sin\alpha_N & 0 \\ 0 & \sin\alpha_N & \cos\alpha_N & 0 \\ 0 & 0 & 0 & 1 \end{bmatrix} = \mathbf{I}_{4\times 4}.$$

Similarly, the forth column and first row becomes:

$$(-1)a_1 + (-1)^2 a_2 + \cdots + (-1)^i a_i + \cdots + (-1)^N a_N = 0.$$

Reorganize the equation will give us:

$$a_1 + a_3 + \cdots + a_{N-1} = a_2 + a_4 + \cdots + a_N.$$

Another way to write the necessary condition is:

$$\sum_i (-1)^i a_i = 0.$$

For a more visual interpretation, when all creases are fully folded, the local axis x_i will alternate direction every time we move from one thick panel to the next thick panel (see Fig. S4 lower right). This alternation is represented using the term $(-1)^i$ in the compact equation.

Equation S4 and S7 provide a useful tool to study the developability and flat foldability of thick origami vertices. These two equations are scalar equations so they are easier to solve when compared to the loop closure constraint. We will studied vertices from degree 4 to degree 10 using these two equations later in this supplementary text.

S2.3 Uniform thickness and other constraints

For a general thick origami vertex, the thickness offset needs to be calculated based on the extrusion or cut in the panels. However, for uniformly thick origami with thickness t, we can provide an equation to directly calculate the offset:

$$\begin{cases} a_i = t, & \text{if } C_i = -1,\ C_{i+1} = 1 \\ a_i = -t, & \text{if } C_i = 1,\ C_{i+1} = -1 \\ a_i = 0, & \text{if } C_i = C_{i+1} \end{cases} \tag{S8}$$

where the term C_i is based on the mountain-valley fold assignment. $C_i = -1$ means the fold is a mountain fold and it folds downward with $\phi_i < 0$ (blue hinge in Fig. S4), and $C_i = 1$ means the fold is a valley fold and folds upwards with $\phi_i > 0$ (red hinge in Fig. S4).

Next, we want to study other constraints for thick origami vertices. We want to first take a look at the DH matrix. The DH matrix has a special structure, where the top left three by three

matrix is a rotation matrix, and the top right three by one vector is a displacement vector. Thus, the matrix can be expressed as:

$$\mathbf{T_i} = \begin{bmatrix} \mathbf{R^i_{3\times3}} & \mathbf{d^i_{3\times1}} \\ \mathbf{0_{1\times3}} & 1 \end{bmatrix}.$$

If we multiply all the DH matrix in the loop closure condition, we will find that the structure of the resultant matrix is unchanged and obtain the following result:

$$\mathbf{T_1 T_2} \dots \mathbf{T_n} = \begin{bmatrix} \mathbf{R_{3\times3}} & \mathbf{d_{3\times1}} \\ \mathbf{0_{1\times3}} & 1 \end{bmatrix} = \mathbf{I_{4\times4}}.$$

This equation can then be split into two equations:

$$\mathbf{R_{3\times3}} = \mathbf{I_{3\times3}} \text{ and } \mathbf{d_{3\times1}} = \mathbf{0_{3\times1}}.$$

The first equation $\mathbf{R_{3\times3}} = \mathbf{I_{3\times3}}$ gives the rotational relationship and is the same loop closure condition as that for thin origami systems. The second equation $\mathbf{d_{3\times1}} = \mathbf{0_{3\times1}}$ considers the translational motion related to thicknesses. In general, the rotational relationship gives three constraining equations after linearization and the translational relationship gives three more equations. Therefore, each thick origami vertex tends to have $N - 6$ degrees-of-freedom (DOFs) while a thin origami vertex has $N - 3$ DOFs, where N is the number of creases.

Another way to show that the thick origami vertex has $N - 6$ DOFs is by directly counting the DOFs of the system. Each panel is a rigid body in 3D space, so it has 6 DOFs. Each rotational hinge in the thick origami will restrict 5 DOFs. Thus, the total DOFs of a thick origami vertex is $N(6 - 5) = N$, which includes the rigid body rotations and translations. If we take out the rigid body rotations and translations, the vertex should have $N - 6$ DOFs.

Therefore, thick origami vertices with $N \geq 8$ hinges have multi-degree-of-freedom (MDOF) kinematics. For over constrained thick origami with $N \leq 6$ rotational hinges, the foldability relies on having redundant constraints. In this case, we have to check the null space of the Jacobian of the flattened constraint equation $\nabla_{\boldsymbol{\phi}}\text{flat}(\mathbf{F}(\boldsymbol{\phi}))$. If there exist a non-trivial solution $\boldsymbol{\phi}(s)$ such that we have:

$$\dim\left(\text{null}\left(\nabla_{\boldsymbol{\phi}}\text{flat}(\mathbf{F}(\boldsymbol{\phi}))\right)\right) = 1,$$

then the origami can fold along the kinematic path $\boldsymbol{\phi}(s)$ with SDOF kinematics. The flat() function converts the $\mathbf{F}$ matrix to a vector. Similar approaches have also been used to study the kinematics of thin origami structures [27].

S3 Developable, Flat Foldable, and Uniformly Thick Vertices

This section demonstrates how we found a vertex to be developable, flat foldable and uniformly thick. We will focus on degree-four to degree-ten vertices. In general, we know that degree-five vertex, degree-seven, and degree-nine vertex do not satisfy flat-foldability conditions (Eqn. S6). Therefore, we will focus on degree-four, degree-six, degree-eight, and degree-ten vertex.

S3.1 Degree-Four Vertices

We start with a degree-four vertex. If a thick degree-four vertex is flat foldable, there is only one possible mountain valley assignment and that is MMMV. Please note that MMMV is equivalent to MMVM, VMMM, and MVMM due to rotational symmetry. Also, switching mountain and valley does not affects the pattern so VVVM is equivalent to MMMV. For the same origami pattern, if looking from top down gives us VVVM mountain valley assignments, looking from bottom up gives us the MMMV assignments. Without loss of generality, we can study the case of MVVV, which gives the following fold angle sign:

$$\phi_1 > 0;\ \phi_2 < 0;\ \phi_3 < 0;\ \phi_4 < 0;$$

Then, for the vertex to satisfy the thickness condition for developability and uniform thickness (Eqn. S4, Eqn. S8), we must have:

$$a_1 = -t; a_2 = 0; a_3 = 0; a_4 = t$$

To further satisfy flat foldability condition (Eqn. S7), we need:

$$(-1)^1(-t) + (-1)^4 t = 2t = 0.$$

This equation shows that we end up having a degenerate solution where $t = 0$. In other words, only a thin origami with zero thickness can satisfy the above conditions. Therefore, we know that degree-four vertex cannot be used to build the MUTOIS that is developable, flat foldable, and has finite uniform thickness.

S2.2 Degree-Six Vertices

First, we can identify distinct mountain valley assignments in degree six vertices, and there are three distinct assignments: MMVVVV, MVMVVV, and MVVMVV. The assignment MVMVVV is the waterbomb vertex and the assignment MVVMVV is the diamond shape vertex. Both vertices were studied in the previous research [1].

A). MVMVVV

We first show that the waterbomb arrangement does not satisfy all conditions for an origami to have developability, flat-foldability, and uniform thickness. For a vertex with a mountain valley assignment like MVMVVV, we have:

$$\phi_1 < 0;\ \phi_2 > 0;\ \phi_3 < 0;\ \phi_4 > 0;\ \ \phi_5 > 0;\ \phi_6 > 0.$$

Then, for the vertex to satisfy the developability (Eqn. S4) and uniform thickness condition (Eqn. S8), we must have:

$$a_1 = t; a_2 = -t; a_3 = t; a_4 = -t; a_5 = 0;\ a_6 = 0.$$

Furthermore, for the vertex to be flat foldable (Eqn. S7), we need to have:

$$(-1)^1(t) + (-1)^2(-t) + (-1)^3(t) + (-1)^4(-t) = -4t = 0.$$

The above equation also requires us to have a degenerate solution with $t = 0$. Therefore, the waterbomb vertex mountain valley assignment cannot produce a thick origami vertex that has uniform thickness and is flat foldable.

B). MVVMVV

Next, we show that the diamond shape vertex can satisfy all conditions. For a vertex with a mountain valley assignment like MVVMVV, we have:

$$\phi_1 < 0;\ \phi_2 > 0;\ \phi_3 > 0;\ \phi_4 < 0;\ \ \phi_5 > 0;\ \phi_6 > 0.$$

Then, for the vertex to satisfy the developable (Eqn. S4) and uniform thickness condition (Eqn. S8), we must have:

$$a_1 = t; a_2 = 0; a_3 = -t; a_4 = t; a_5 = 0;\ a_6 = -t.$$

Furthermore, for the vertex to be flat foldable (Eqn. S7), we need:

$$(-1)^1(t) + (-1)^3(-t) + (-1)^4(t) + (-1)^6(-t) = 0.$$

This shows that the diamond shape vertex indeed satisfies the thickness-based necessary conditions for flat-foldability, developability, and uniform thickness. Finally, we need to further ensure that the solution can also satisfy the remaining necessary conditions in Table S1. This diamond pattern vertex can have the following sector angle assignment:

$$\alpha_1 = \alpha_4 = \pi - 2\alpha_2 \text{ and } \alpha_2 = \alpha_3 = \alpha_5 = \alpha_6,$$

and the assigned sector angles do satisfy sector angle requirements for developability and flat foldability (Eqn. S3 and Eqn. S5). The nontrivial folding motion of this thick origami vertex is found in [1]:

$$\phi_1 = \phi_4;\quad \phi_2 = \ \phi_3 = \phi_5 = \phi_6;$$

$$\tan\frac{\phi_1}{2} = -\cos\alpha_1 \tan\frac{\phi_2}{2}.$$

For the proposed MUTOIS, we set:

$$\alpha_1 = \alpha_4 = \frac{\pi}{2} \text{ and } \alpha_2 = \alpha_3 = \alpha_5 = \alpha_6 = \frac{\pi}{4}.$$

C). MMVVVV

The mountain valley assignment MMVVVV is not a widely studied vertex even in literature on thin origami. This is probably because popular origami tessellations are not based on this vertex. The thin version of MMVVVV vertex exists and have three degrees-of-freedom. We can show that uniformly thick MMVVVV vertex does not exist by further considering panel penetrations.

For a vertex with a mountain valley assignment like MMVVVV, we have:

$$\phi_1 < 0;\ \phi_2 < 0;\ \phi_3 > 0;\ \phi_4 > 0;\ \ \phi_5 > 0;\ \phi_6 > 0.$$

Then, for the vertex to satisfy the developability (Eqn. S4) and uniform thickness condition (Eqn. S8), we must have:

$$a_1 = 0; a_2 = t; a_3 = 0; a_4 = 0; a_5 = 0;\ a_6 = -t.$$

Furthermore, for the vertex to be flat foldable (Eqn. S7), we need to have:

$$(-1)^2(t) + (-1)^6(-t) = 0.$$

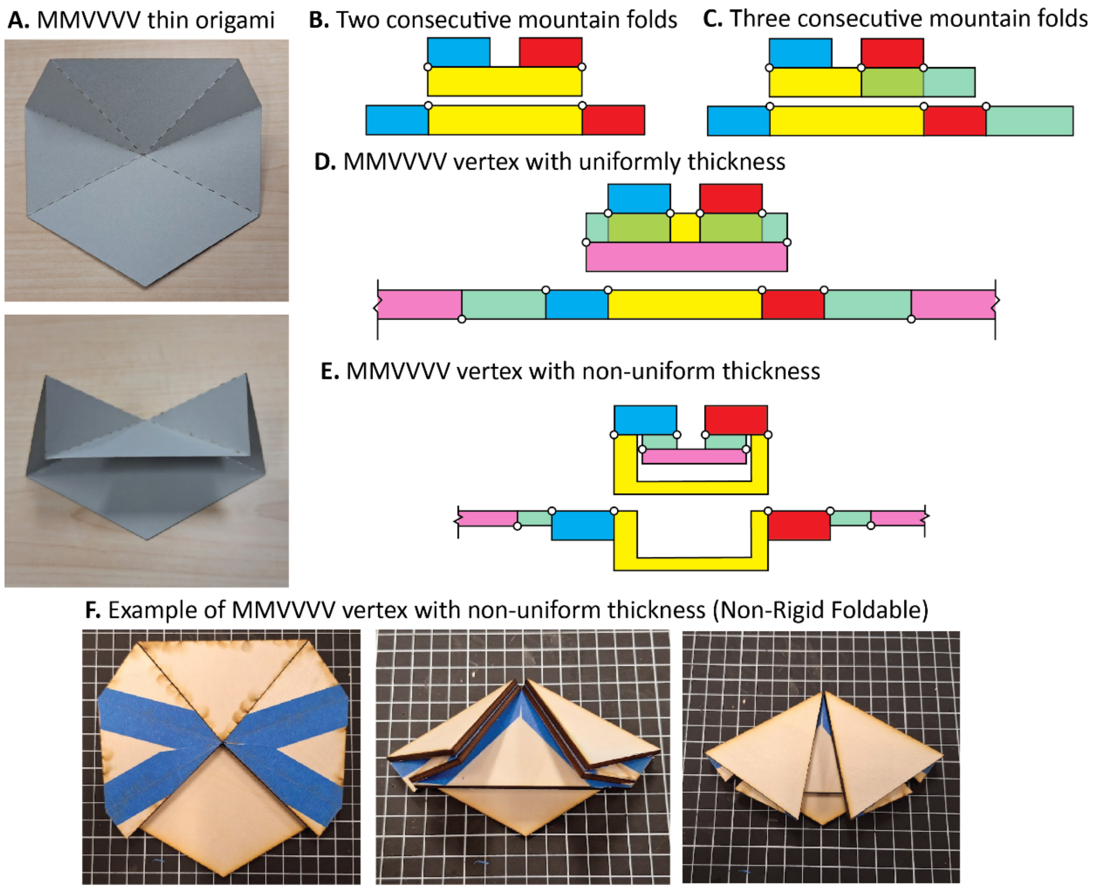

Figure S6. Study on the MMVVVV vertex.

Although we can satisfy the thickness constraints for developability, flat foldability, and uniformly thickness, such thick origami vertex still does not exist because having four consecutive valley folds creates panel penetration. For uniformly thick origami vertices, having more than two consecutive mountain folds or valley folds will produce overlapping panels as shown in Fig. S6C and S6D – this is not physically possible. However, it is possible to avoid penetration if we allow panels to have extruded parts or cuts as shown in Fig. S6E and Fig. S6F. This example shows one limitation of the derived conditions – they are necessary conditions without considering panel penetration.

S2.3 Degree-Eight Vertices

As a demonstration, we want to use the proposed new sign convention and thickness conditions to show that degree-eight vertices are not developable, flat foldable, and uniformly thick (we remove the requirement that the vertex is SDOF). With this derivation, we want to further highlight the usefulness of the new sign convention and the derived thickness conditions. For degree-eight vertex, we need five mountain folds and three valley folds to satisfy the Maekawa-Justin theorem. Thus, the possible mountain valley assignments include A) MMMMMVVV; B) MMMMVMVV; C) MMMVMMVV; D) MMMVMVMV; E) MMVMVMMV.

A) MMMMMVVV

In this case, folding angles need to be:

$$\phi_1 < 0;\ \phi_2 < 0;\ \phi_3 < 0;\ \phi_4 < 0;\ \ \phi_5 < 0;\ \phi_6 > 0;\ \phi_7 > 0;\ \phi_8 > 0.$$

Then, for the vertex to satisfy the developable (Eqn. S4) and uniform thickness condition (Eqn. S8), we must have:

$$a_1 = 0; a_2 = 0; a_3 = 0; a_4 = 0; a_5 = t;\ a_6 = 0;\ a_7 = 0;\ a_8 = -t.$$

Furthermore, for the vertex to be flat foldable (Eqn. S7), we need to have:

$$(-1)^5(t) + (-1)^8(-t) = -2t = 0.$$

Thus, this vertex cannot have non-zero thickness.

B) MMMMVMVV

In this case, folding angles need to be:

$$\phi_1 < 0;\ \phi_2 < 0;\ \phi_3 < 0;\ \phi_4 < 0;\ \ \phi_5 > 0;\ \phi_6 < 0;\ \phi_7 > 0;\ \phi_8 > 0.$$

Then, for the vertex to satisfy the developable (Eqn. S4) and uniform thickness condition (Eqn. S8), we must have:

$$a_1 = 0; a_2 = 0; a_3 = 0; a_4 = t; a_5 = -t;\ a_6 = t;\ a_7 = 0;\ a_8 = -t.$$

Furthermore, for the vertex to be flat foldable (Eqn. S7), we need to have:

$$(-1)^4(t) + (-1)^5(-t) + (-1)^6(t) + (-1)^8(-t) = 2t = 0.$$

Thus, this vertex cannot have non-zero thickness.

C) MMMVMMVV

In this case, folding angles need to be:

$$\phi_1 < 0;\ \phi_2 < 0;\ \phi_3 < 0;\ \phi_4 > 0;\ \ \phi_5 < 0;\ \phi_6 < 0;\ \phi_7 > 0;\ \phi_8 > 0.$$

Then, for the vertex to satisfy the developable (Eqn. S4) and uniform thickness condition (Eqn. S8), we must have:

$$a_1 = 0; a_2 = 0; a_3 = t; a_4 = -t; a_5 = 0;\ a_6 = t;\ a_7 = 0;\ a_8 = -t.$$

Furthermore, for the vertex to be flat foldable (Eqn. S7), we need to have:

$$(-1)^3(t) + (-1)^4(-t) + (-1)^6(t) + (-1)^8(-t) = -2t = 0.$$

Thus, this vertex cannot have non-zero thickness.

D) MMMVMVMV

In this case, folding angles need to be:

$$\phi_1 < 0;\ \phi_2 < 0;\ \phi_3 < 0;\ \phi_4 > 0;\ \ \phi_5 < 0;\ \phi_6 > 0;\ \phi_7 < 0;\ \phi_8 > 0.$$

Then, for the vertex to satisfy the developable (Eqn. S4) and uniform thickness condition (Eqn. S8 and), we must have:

$$a_1 = 0; a_2 = 0; a_3 = t; a_4 = -t; a_5 = t;\ a_6 = -t;\ a_7 = t;\ a_8 = -t.$$

Furthermore, for the vertex to be flat foldable (Eqn. S7), we need to have:

$$(-1)^3(t) + (-1)^4(-t) + (-1)^5(t) + (-1)^6(-t) + (-1)^7(t) + (-1)^8(-t) = -6t = 0.$$

Thus, this vertex cannot have non-zero thickness.

E) MMVMVMMV

In this case, folding angles need to be:

$$\phi_1 < 0;\ \phi_2 < 0;\ \phi_3 > 0;\ \phi_4 < 0;\ \ \phi_5 > 0;\ \phi_6 < 0;\ \phi_7 < 0;\ \phi_8 > 0.$$

Then, for the vertex to satisfy the developable (Eqn. S4) and uniform thickness condition (Eqn. S8 and), we must have:

$$a_1 = 0; a_2 = t; a_3 = -t; a_4 = t; a_5 = -t;\ a_6 = 0;\ a_7 = t;\ a_8 = -t.$$

Furthermore, for the vertex to be flat foldable (Eqn. S7), we need to have:

$$(-1)^2(t) + (-1)^3(-t) + (-1)^4(t) + (-1)^5(-t) + (-1)^7(t) + (-1)^8(-t) = 4t = 0.$$

Thus, this vertex cannot have non-zero thickness.

S2.4 Degree-Ten Vertices

Finally, we want to show that there is a degree-ten vertex that is developable, flat-foldable, and uniformly thick. The solution will have a mountain valley assignment of MMVMVMMVMV. In this case the fold angles have signs as:

$$\phi_1 < 0;\ \phi_2 < 0;\ \phi_3 > 0;\ \phi_4 < 0;\ \ \phi_5 > 0;\ \phi_6 < 0;\ \phi_7 < 0;\ \phi_8 > 0;\ \phi_9 < 0;\ \phi_{10} > 0.$$

Then, for the vertex to satisfy the developable (Eqn. S4) and uniform thickness condition (Eqn. S8 and), we must have:

$$a_1 = 0; a_2 = t; a_3 = -t; a_4 = t; a_5 = -t;\ a_6 = 0;\ a_7 = t;\ a_8 = -t, a_9 = t;\ a_{10} = -t.$$

Furthermore, for the vertex to be flat foldable (Eqn. S7), we need to have:

$$(-1)^2(t) + (-1)^3(-t) + (-1)^4(t) + (-1)^5(-t) + (-1)^7(t) +$$

$$(-1)^8(-t) + (-1)^9(t) + (-1)^{10}(-t) = 0.$$

With all terms canceled with each other, we know that this vertex can satisfy all the required thickness conditions. The following Fig. S7 shows a simulated degree-10 vertex based on the above derivation (implemented with the Supplementary Code 1 with the file name "SingleVertex_D10_Kinematic_Analysis.m"). Although this vertex can satisfy all the thickness conditions, it has 4 DOFs, which makes its deployment difficult to control. In general, solving the loop closure conditions for this 4-DOF vertex can be highly challenging. However, the proposed thickness condition provides a fast and easy way to investigate the characteristics of this MDOF degree-10 vertex, which highlight the usefulness of the proposed thickness conditions.

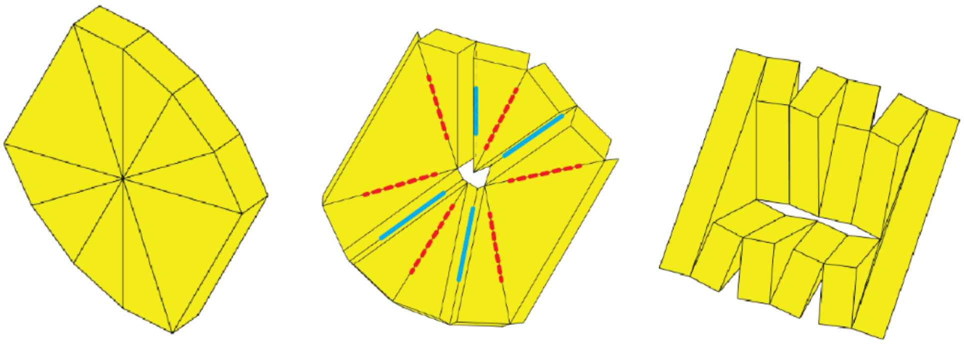

Figure S7. A developable, flat-foldable, and uniformly thick degree-10 vertex.

S4 Locking Devices and Digital Fabrication for MUTOIS

In this work, the origami deployment is controlled by strategically locking hinges and lifting the thick origami at appropriate locations. The main text briefly introduced four different locking devices proposed for the MUTOIS. Here, we focus on those three locking devices that are fabricated and used in this work (see Fig. S8). Figure S8A shows a sliding lock design, which has a slider at the back side. When the slider is closed, it prevents the hinge from rotating. Figure S8B shows a latch lock design that can close the hinge by closing the latch. Finally, Fig. S8C shows a gusset plate connection. When the gusset plate is installed, the hinge cannot rotate. Supplementary Movie 2 further shows how to use these locking devices.

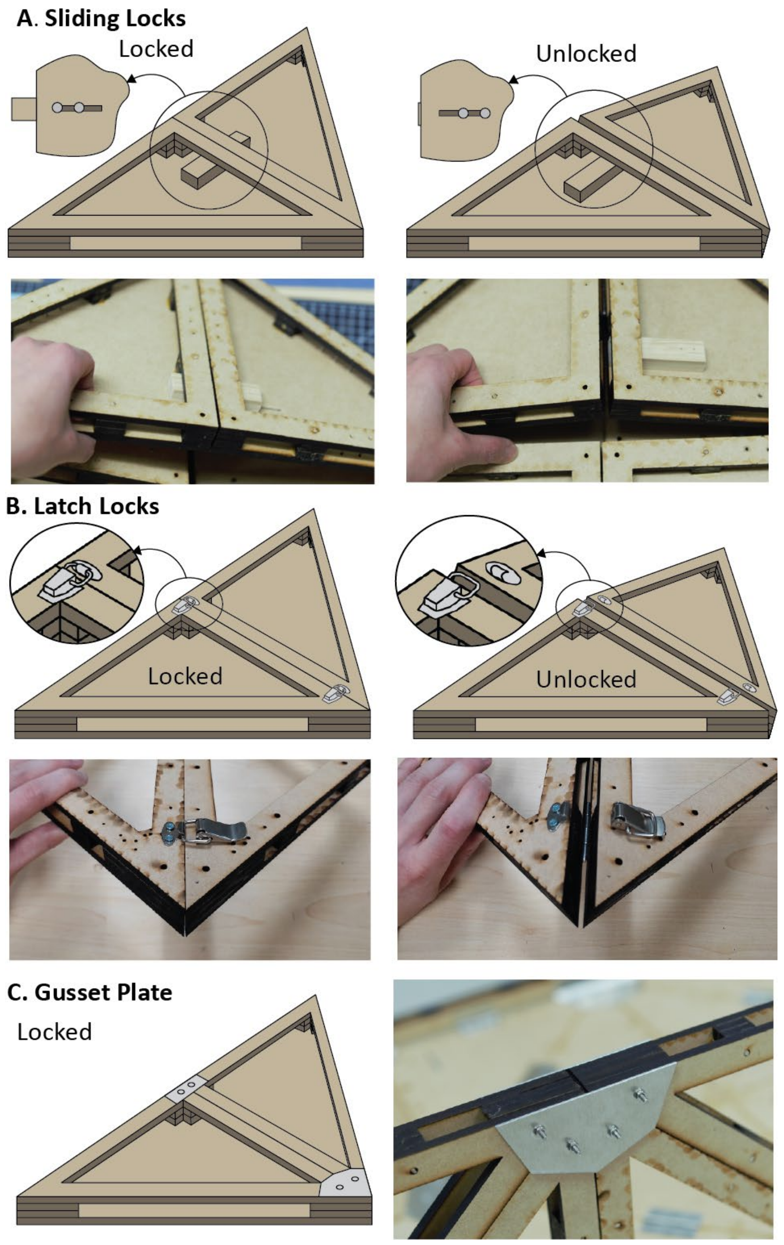

Figure S8. Locking devices for thick origami systems.

Among these three forms of locking devices, sliding locks and latch locks allow for rapid deployment. Both mechanisms can be rapidly closed to stop the rotation of selected hinges. These devices are preferred if speed is more important for the application. Although these two locking devices can support a considerable amount of load, they are not designed to achieve a high load-carrying capacity. On the other hand, gusset plates provide improved loading capacity but require longer time to install. Therefore, these connectors are preferred if a large load carrying capacity is needed. In the future, dedicated locking devices with fast locking and high capacity can be developed for thick origami systems (such as the self-latching locks in Fig. 3).

Figure S9 shows a digital fabrication process developed to build MUTOIS. We use mid-density fiber (MDF) board to build MUTOIS because it is inexpensive, lightweight, and has relatively uniform material properties. First, the design of thick origami structure is drafted into a CAD software program. Then, panels are cut out from MDF boards using laser cutter. A Universal Laser System laser cutter is used to cut the 0.25 inch thick MDF board. The cutting is performed using 60% power and 2% speed setup, which is enough to cut through the material without burning it. When working with laser cutting, we noticed that not all MDF boards can be successfully cut. There are multiple brands of MDF products available on the market, but from our experience, only those made with soft wood (e.g. pine) can be cut out nicely.

When designing the CAD drawings, screw holes are included so that better assembly accuracy can be achieved. Thick origami panels are built with multiple layers of MDF board, and these different layers are glued together with wood glue. Next, these panels are connected using rotational hinges, which are smaller door hinge like products for DIY and craft projects. The proposed fabrication process is inexpensive and can be extended to larger scales and stiffer structural materials like glulam and structural steel.

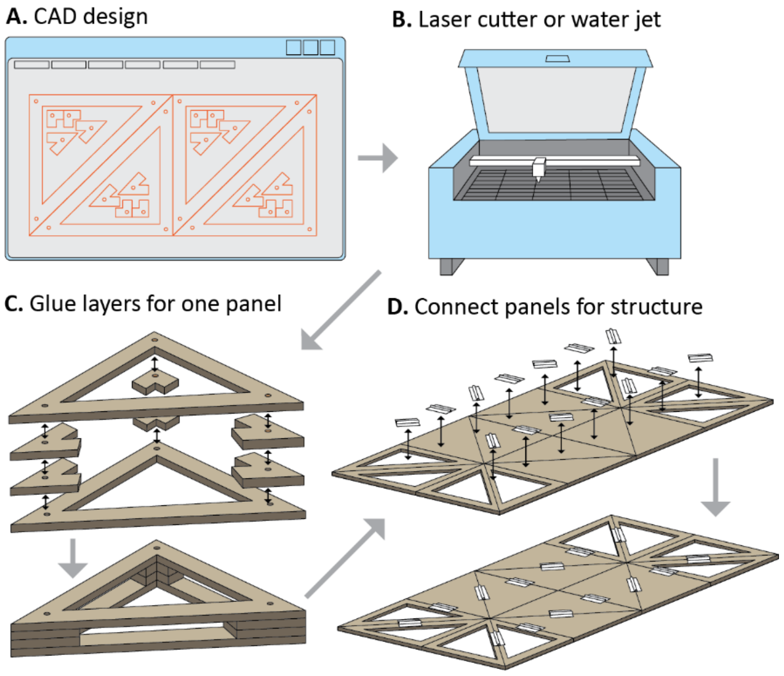

Figure S9. Fabrication process of the MUTOIS system.

S5. Bar and Hinge Simulation for Thick Origami

Traditional kinematic analysis cannot capture load-carrying behaviors of MUTOIS while traditional mechanical simulations are not suitable for capturing large-deformation deployment. To resolve this challenge, we develop a bar and hinge simulation with a new formulation tailored for thick panels and connectors to simulate both deployment and load-carrying behaviors of MUTOIS. Inside a bar and hinge model, the origami geometry is represented using bar elements and rotational spring elements (see Fig. S10A) [28, 29]. The bar elements capture stretching and shearing in the panels, and the rotational spring elements capture rotations about crease lines. In our new formulation, thick origami panels (grey bars in Fig. S610) and connectors (black bars in Fig. S610) are modeled separately, allowing us to represent different stiffness in hinges and panels.

There are two types of panels within the thick origami bridge shown in Fig. 6 and Fig. 7 of the manuscript: a solid panel and a truss panel. The bar areas for these two panels need to be set differently. The cross section of the truss panel (for the MUTOIS bridge tested in Fig. 7) is shown in the Fig. S10C and has an area of about $0.4in^2 = 0.000258m^2$. For truss-like panels, we set $A_1 = 0.4in^2/3 = 0.000258m^2/3$ because three bars contribute to the total cross section. Connector bar areas are assumed to have $A_2 = A_1/5$ and $A_3 = A_1$. The area of A_2 bars are taken as 1/5 of the original bar areas to consider the relatively soft shearing behaviors between the panels. The bar areas for solid panels are taken as 3 times of the truss-like panels, because solid panels have larger stiffness than the truss panels. The Young's modulus of MDF material is taken as 3.2 GPa based on the material testing results obtained in section S10. With these area assignments, the bar and hinge model can capture the linear load bearing behaviors of the MUTOIS bridge reasonably well.

The hinge stiffness is set to be a small value (0.000001 N/m) for unlocked and free-to-rotate hinges and the stiffness is set to be a large value for the locked hinges (100,000 times larger). Using a small hinge stiffness rather than a zero hinge stiffness can avoid numerical instability when using the Newton-Raphson method to track the equilibrium of the full system. To simulate the behaviors of locking creases for load bearing capability, additional bar elements are added to represent gusset plates.

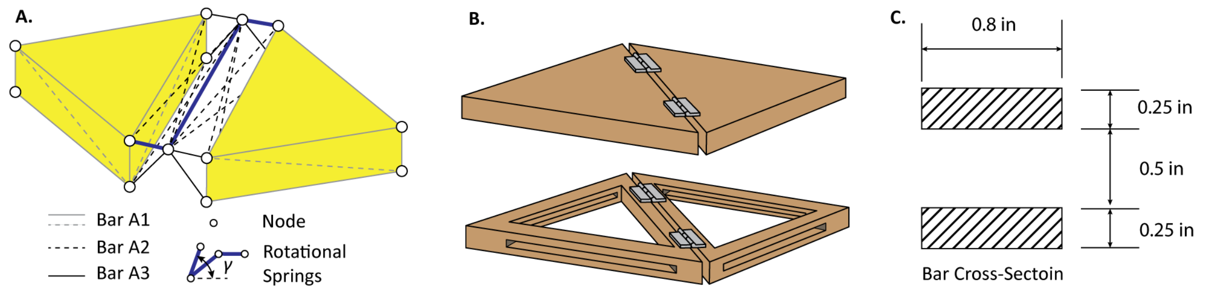

Figure S10. Bar and hinge simulation for thick origami.

The file "ModularOri_FullBridge.m" in the Supplementary Code 1 simulates the full deployment and load-carrying process of the MUTOIS bridge (simulation in Fig. 6 and Fig. 7 of the manuscript). In this simulation, the MUTOIS bridge is first folded from flat to a compact

state for transportation. Then, the MUTOIS bridge consecutively deploys to a flat configuration and then a bridge configuration. Finally, after locking the creases at a bridge state, the simulation can capture load-carrying behaviors of this MUTOIS bridge. The load-displacement curves and the load-strain curves are shown in Fig. 7 of the manuscript. This new bar and hinge formulation provides a useful tool to study deployable load-carrying structures, because it captures both kinematic and load-carrying behaviors.

Next, we show that the bar and hinge model can capture identical bifurcation occurrence when compared with traditional kinematic analysis. Traditional kinematic analysis finds the bifurcation of a thick origami vertex by checking its kinematic constraint function:

$$\mathbf{F}(\boldsymbol{\phi}) = \mathbf{T_1 T_2} \dots \mathbf{T_6} - \mathbf{I}_{4\times 4} = \mathbf{0}_{4\times 4}.$$

We can first eliminate the final row in the four-by-four matrix (which is constant) and flatten the matrix into a 12 by 1 vector. We then take the derivative of this 12 by 1 vector with respect to the six folding angles (to calculate $\nabla_{\boldsymbol{\phi}}\text{flat}(\mathbf{F}(\boldsymbol{\phi}))$) and obtain a 12 by 6 Jacobian matrix $\mathbf{C}$. The null space of this $\mathbf{C}$ matrix represents the infinitesimal kinematically admissible folding motion.

By tracing the singular value of this $\mathbf{C}$ matrix, we can study the bifurcation of this thick origami vertex. A kinematically admissible folding path $\boldsymbol{\phi}(s)$ will have a singular value of zero at any point ($s = s_i$). If the null space of $\mathbf{C}$ matrix has one dimension – there is only one folding path $\boldsymbol{\phi}(s)$ with a singular value of zero – we know that the kinematic folding path has a SDOF kinematics. If there are some discontinuous points along the path $\boldsymbol{\phi}(s)$ that have more than one singular value to be zero, these points are called bifurcation points or multifurcation points. In general, the developed configuration (flat state) of an origami system is where kinematic bifurcation happens.

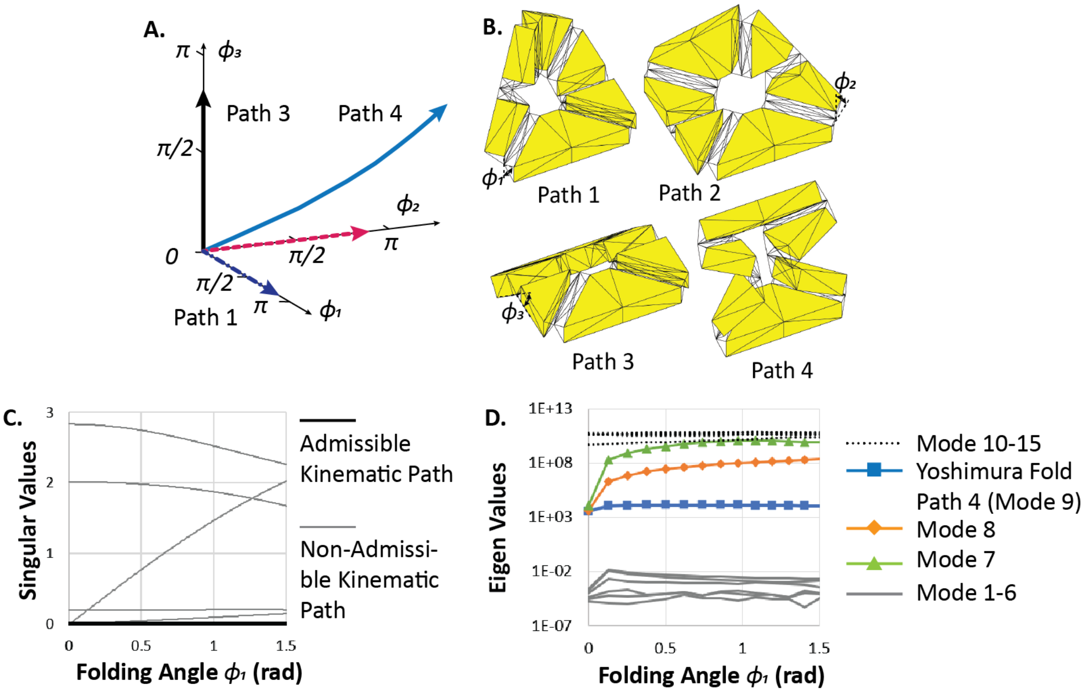

Figure S11. Kinematic bifurcation of single origami vertex.

Figure S11A and S11B show four kinematic folding paths of a thick MUTOIS vertex. In addition to the non-trivial Yoshimura folding path (path 4), there are three degenerate folding paths. These paths exist because there are three collinear creases around this degree-six vertex. Figure S11C further shows how the singular value changes as the thick origami vertex folds along the Yoshimura folding path (path 4). At $\boldsymbol{\phi}(s) = 0$, we have a bifurcation point where all four kinematic folding paths meet each other. However, as we fold away from the flat configuration, only the Yoshimura path (path 4) will show a singular value of zero. This analysis is done using the Supplementary Code 1 (readers are referred to the following file named "SingleVertex_Kinematic_Bifurcation_Analysis.m").

The same bifurcation behavior can be captured using the bar and hinge simulation as shown in Fig. S11B and Fig. S11D. In a bar and hinge model, folding modes can be investigated using eigen values of the stiffness matrix $\mathbf{K}(\mathbf{x})$, where $\mathbf{x}$ is a vector representing the nodal coordinates of the origami. The first six modes of the stiffness matrix are correlated with the rigid body motions (including three translations and three rotations). Next, when the vertex is at the developed flat state, we find that modes 7 through 9 have relatively small eigen values. These three modes are correlated with the four folding paths in the MUTOIS vertex. Mode 10 and larger modes are associated with high energy deformations where panels deform. As we fold away from the flat configuration, two of the three folding modes will no longer be kinematically admissible (panel deformation occurs), and their eigenvalues will become large quickly. This is similar to what we see in the singular value analysis shown in Fig. S11C.

This case study shows that the bar and hinge model can capture identical bifurcation occurrence as kinematic analysis. However, we should point out that both singular value analysis and eigen value analysis cannot predict which kinematic folding path a MUTOIS will deform into under a specific scenario with given boundary condition and force application. To capture which path the structure will deform into, we need to set up nonlinear equilibrium analysis using the bar and hinge model, because this analysis can capture the strain energy and the gravitational potential within the MUTOIS. When implementing the nonlinear equilibrium analysis, the applied force is increased incrementally, and the equilibrium is tracked using Newton-Raphson method. This capability or bar and hinge simulation is highlighted using the simulation shown on Fig. 6 in the manuscript, where the simulation can predict accurate folding shapes of MUTOIS with different locked hinges and force application.

Moreover, the bar and hinge model can simulate the load-carrying capacity of MUTOIS, which cannot be done using kinematic simulations. We can use the same Newton-Raphson solver to track the equilibrium of the loading process. Figure 7B and 7C show the simulated load-carrying behaviors of a MUTOIS bridge. The simulation can predict accurate initial stiffness and buckling shapes of the MUTOIS bridge. All simulations are implemented using codes in the Supplementary Code 1.

S6. Construction Details of MUTOIS Bridge

S6.1 MUTO Bridge with Sliding Locks and Gusset Plates

Figure S12 shows detailed designs of the MUTOIS bridge using sliding locks and gusset plates. This design is associated with the Supplementary Movie 6. For this design, panels are connected with each other using a single hinge placed at the center of the crease. When assembling the system to the 3D bridge state, gusset plates are used to further connect origami panels to improve the load carrying capability.

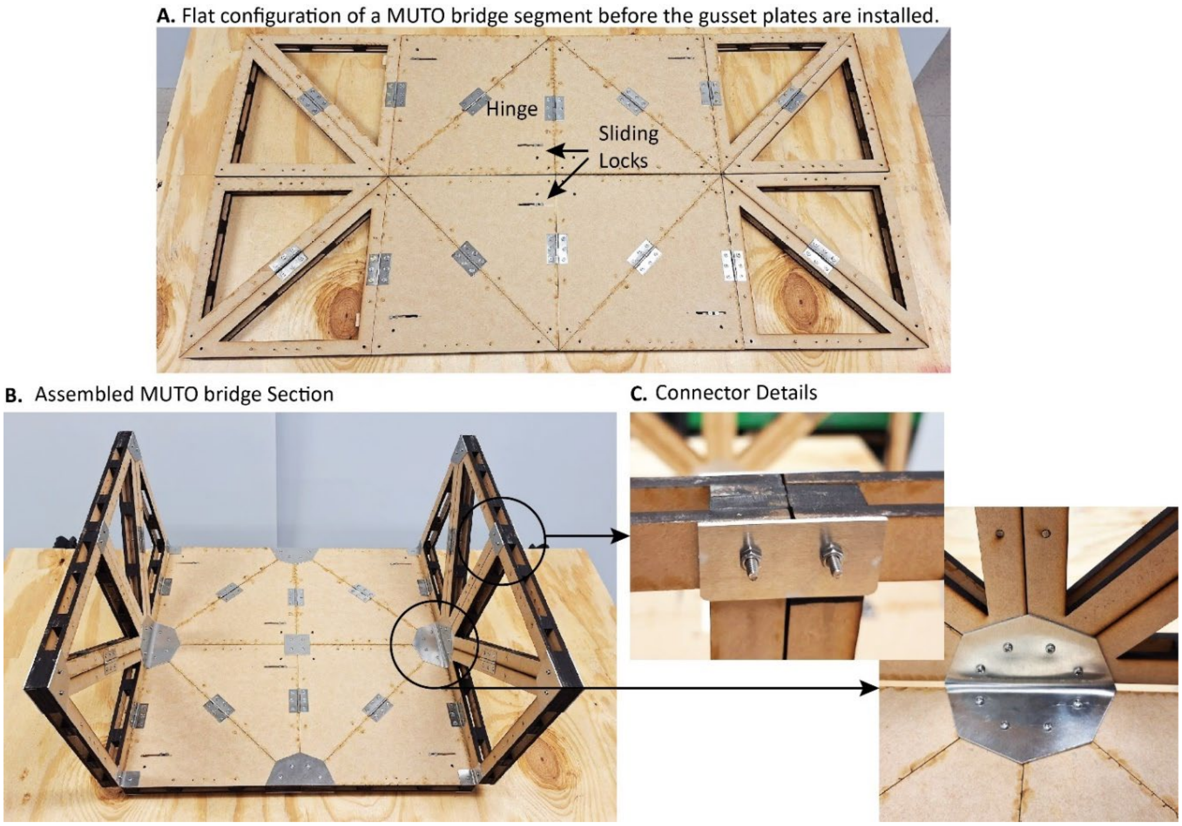

Figure S12. MUTOIS bridge with sliding locks and gusset plates.

In this design, the sliding locks are used to temporarily close selected hinges so that we can control the multi-path folding motion. These sliding locks can be closed from the front side or from the back side of the panels. There are six sliding locks on one section of the MUTOIS bridge design – the black horizontal slots on Fig. S12A are sliding locks.

When assembling this MUTOIS bridge, gusset plates are placed from both sides of MDF panels and connected with M3 bolts (see Fig. S12C). These gusset plates share similar geometry and design with gusset plates used in heavy timber or steel structures. As demonstrated in Supplementary Movie 1 and Supplementary Movie 6, assembling gusset plate is a time-consuming process and may require a large number of labors. Therefore, we designed another MUTOIS bridge using latch locks and we show that these new connectors can speed up the assembly drastically.

S6.2 MUTOIS Bridge with Sliding Locks, Latch Locks, and Gusset Plates

Here we demonstrate construction details of the MUTOIS bridge with sliding locks, latch locks, and gusset plates. This design is used to record the Supplementary Movie 3. The use of latch locks can drastically speed up the assembly speed of MUTOIS.

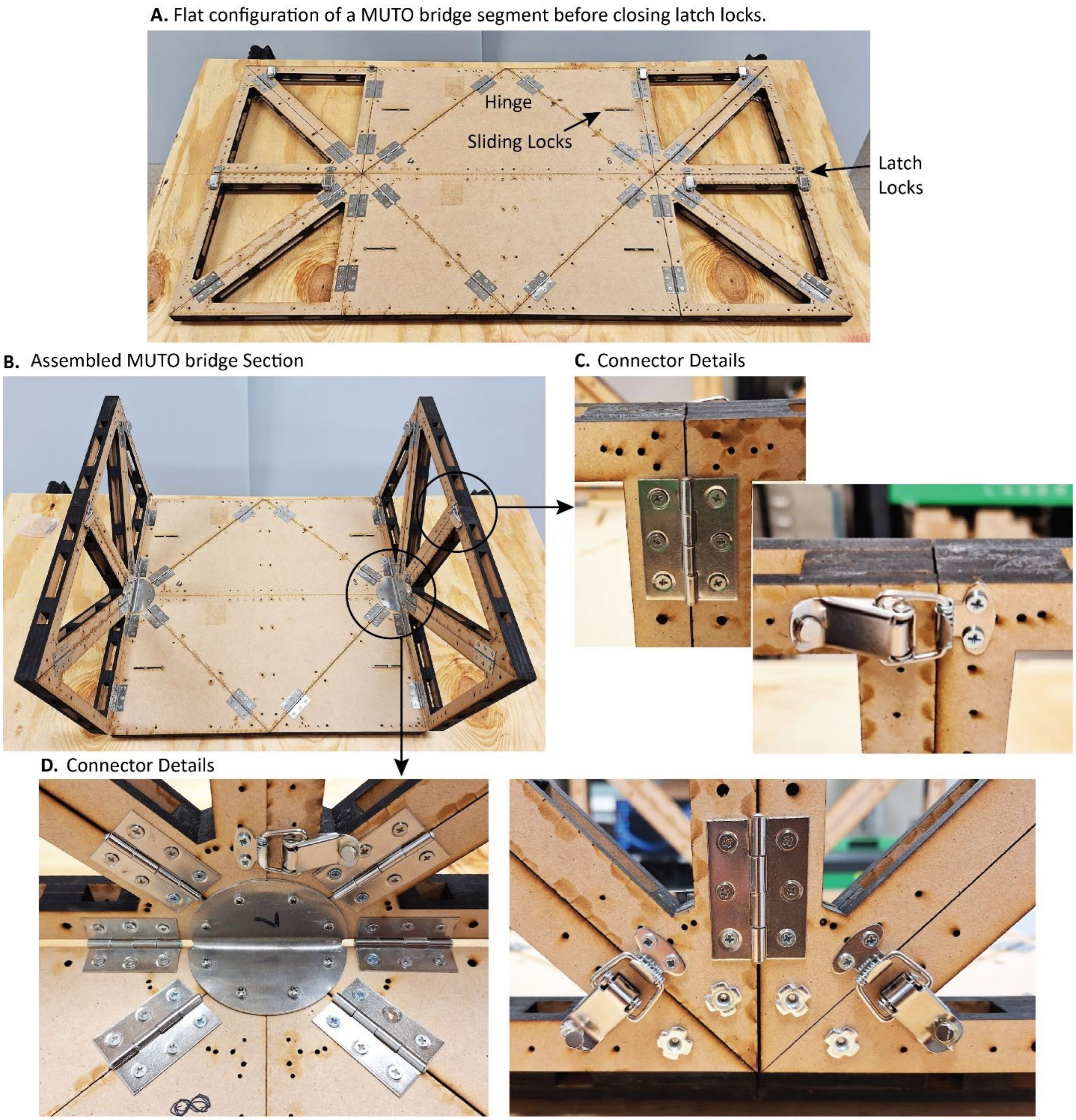

Figure S13. MUTOIS bridge with sliding locks, latch locks, and gusset plates.

Figure S13A shows the layout of the upgraded MUTOIS. In this new design, each crease is connected using two folding hinges rather than one. On the other side of the hinge, there is a

latch lock. When the latch locks are closed, the hinge line cannot fold. Instead, the system can carry a considerably large force through the structural hinges and the latch locks.

We can eliminate one fold line in the center of the MUTOIS bridge as it is not needed for the folding kinematics. Alternatively, we can also keep the fold line so that the structure maintains high modularity for rapid repair or reuse. Figure S13A shows the design without the center fold line. During the assembly process, latch locks will be closed to turn the mechanism into a structure. A small number of gusset plates are still used to form the 90 degree connector as shown in Fig. S13D. In this upgraded design, the gusset plates are assembled from just one side with insert nuts on the other side.

Assembling this new bridge (recorded in Supplementary Movie 3) is much faster than the previous design shown in S6.1 and Supplementary Movie 6. However, the use of latch locks or other rapid assembly locks are not common in existing civil engineering practice. There is limited literature to estimate the capacity of these latch locks so these designs still need future research before they can be used in real engineering practice. However, as demonstrated in Supplementary Movie 3 this fast-assembly MUTOIS bridge can still carry the weight of a person walking across it, showing that the latch locks are strong enough to carry loads.

S7. Cyclic Test of the MUTOIS Bridge with Gusset Plates

Here, we demonstrate details about the cyclic testing of the MUTOIS bridge connected with Gusset Plates (the one shown in section S6.1). Figure S14A shows the experimental setup for the MUTOIS bridge. A three-point bending experiment is performed using an Instron Beam Tester. The end of the bridge is simply supported, and the supporting structures are shown in Fig. S14B and Fig S14C. The side trusses are simply supported at both ends using stacks of 2 by 4 and 2 by 6 lumber. This support can be accurately represented as a simply supported condition that transfers no moment. The MUTOIS bridge in Fig. S14 uses gusset plate connectors for load-carrying (the one shown in section S6.1). Connecting these gusset plates takes about 120 minutes (see Supplementary Movie 6).

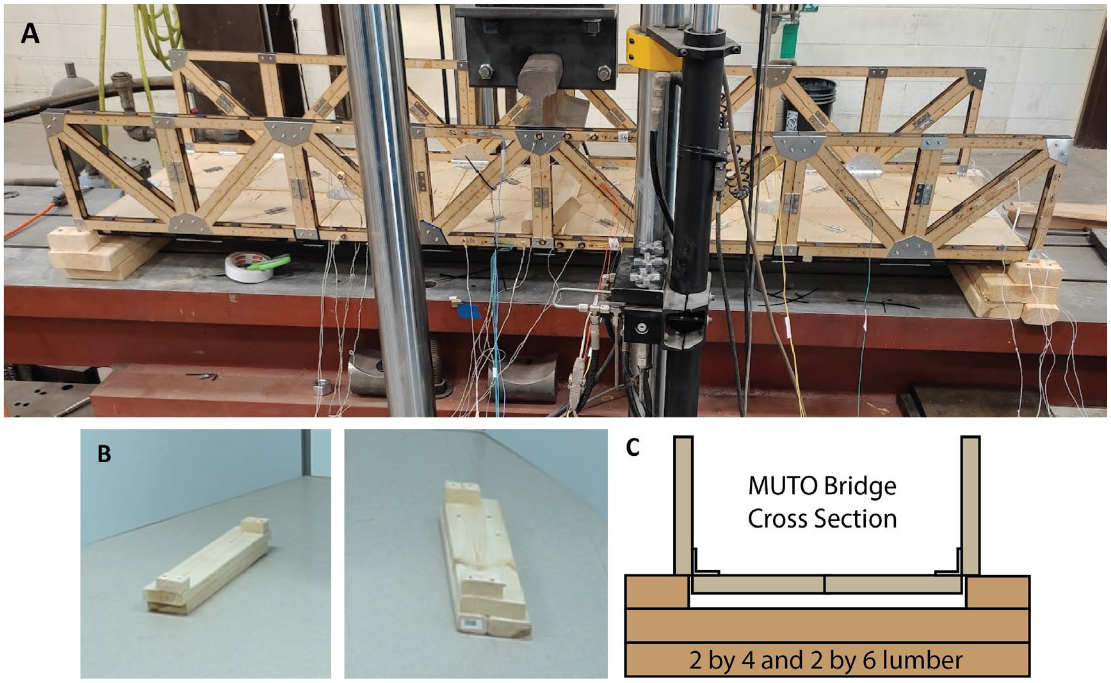

Figure S14. Load carrying experiments of the deployable bridge.

S7.1 Calculation of Connector Strength

Figure S15A shows an analytical solution to find internal forces of the MUTOIS bridge. Assuming that the center of the bridge is loaded with a 2500N force (which is the ultimate load measured from experiment), each side of the truss will support 1250N. This 1250N force is applied to the deck of the bridge near connector 1 on Fig. S15B. Because this truss is structurally determinate, we can find the internal forces as shown on Fig. S15A. Please note that this simplified calculation ignores the contribution of the floor system, which can further reduce the tensile stress within the truss portion of the bridge structure. However, using the internal forces on Fig. S15A to check the strength of connectors on the tensile side (bottom) is conservative, which is why we will use it for the calculations here.

The detailed constructions of these gusset plates are shown in Fig. S15B. Because the center connector 1 sees the largest tension force, we provide a sample calculation to evaluate the

nominal capacity of this plate. This connector 1 undergoes a 2500N tensile force, and the construction of this connector is shown on Fig S15B and S15C. We will first check the tensile force capacity of the MDF and the Aluminum (Al) connection plate (see Fig. S15D). The cross-section area of the MDF at the failure surface is:

$$A_{MDF} = (2cm - 3mm) \times 2.5cm = 4.25cm^2$$

Thus, using the measured MDF material strength of 18MPa, the tensile strength of this cross section can be calculated as:

$$F_{MDF} = A_{MDF} \times \sigma_{MDF} = 7650N > 2500N$$

Therefore, we know that the MDF cross-section is sufficient to support the load.

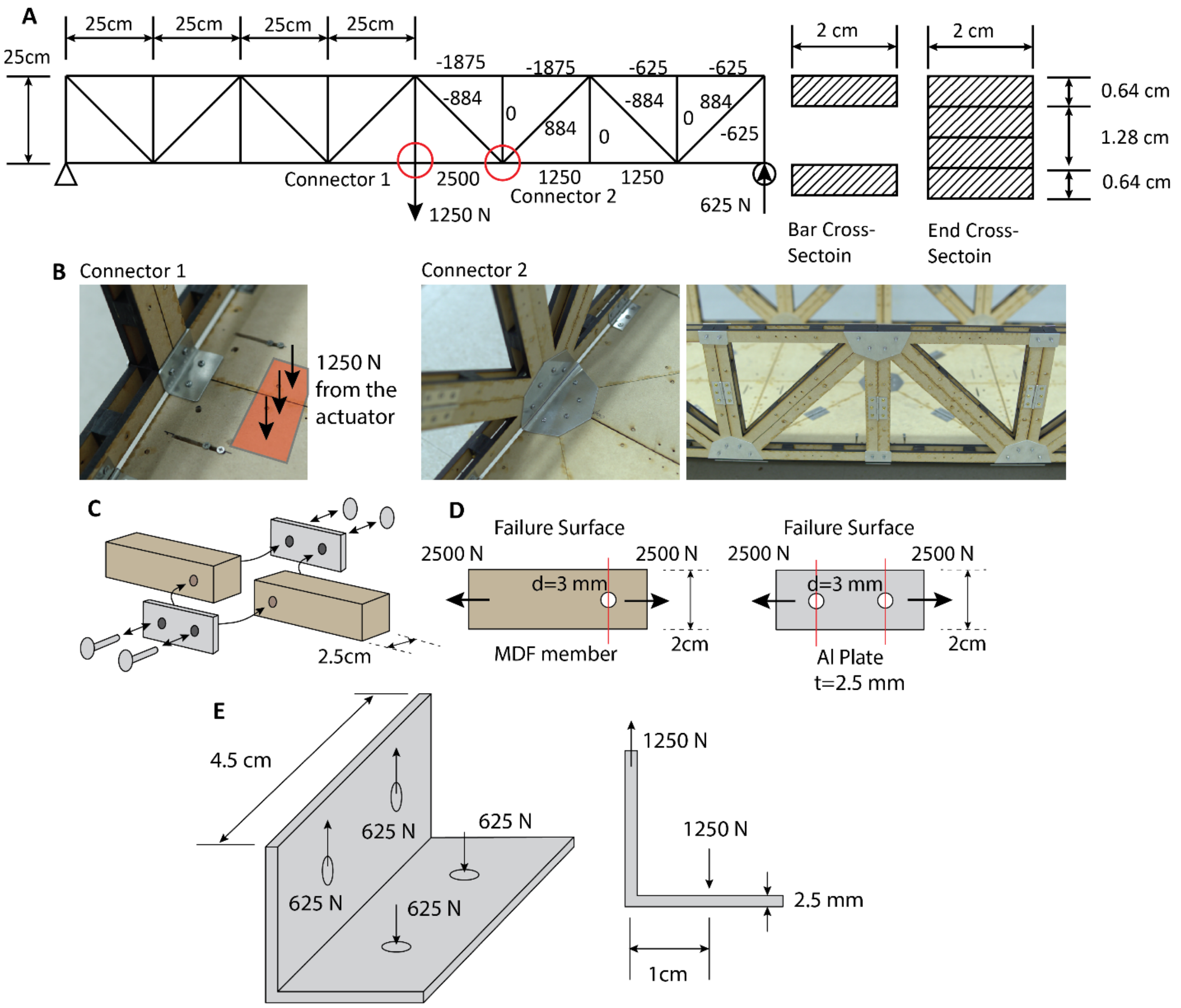

Figure S15. Calculation of the capacity of the MUTOIS bridge and the connectors

Next, we check the strength of the Aluminum (Al) connection plate for this 2500N force. The total cross-section area of the Al plate is:

$$A_{Al} = (2cm - 3mm) \times 2.5mm \times 2 = 0.85cm^2$$

The yield strength of Al (6061) is 276 MPa [30] so the tensile strength of the Al plate is:

$$F_{Al} = A_{Al} \times \sigma_{Al} = 23460N > 2500N$$

Therefore, we know that the Al plate cross-section is sufficient to support the load.

The shear capacity and the bearing capacity of M3 bolts can be directly obtained from the datasheet provided by the manufacturer [31]. The double shear capacity of M3 bolt is:

$$V_{M3} = 3960N > 2500N$$

The bearing capacity of M3 bolt against two 2.5 mm thick Al plate is:

$$V_{M3,5mm\ bearing} = 3800N > 2500N$$

Thus, we know that the bolts can support the ultimate load acting on the bridge.

Finally, we consider the bending moment acting on the angled plate from the loading at the mid span (see Fig. S15E). For this plate, the generated moment from the loading is:

$$M_{applied} = 1cm \times 1250N = 12.5Nm$$

The bending moment capacity of the plate is:

$$M_{strength} = 276MPa \times \frac{4.5cm \times 2.5mm^2}{6} = 12.9Nm > 12.5Nm$$

Thus, the connection plate will not yield at the ultimate load. We should also consider the shear capacity of this plate:

$$V_{strength} = 207MPa \times 4.5cm \times 2.5mm = 23287.5N > 1250N$$

Although the bending moment is close to its capacity, the structure has more capacity to transfer the locally applied forces at the mid span because there are structural hinges next to the connection plate. We have neglected the contribution of these structural hinges in the above calculation, so the calculation is conservative. Moreover, because we are using simple bolts with no prestress, the plate itself can move relative to the bolts due to sliding. This relative motion can further release the bending moment acting on this connector plate.

The above calculation only demonstrates nominal capacity without showing any strength reduction factors. For real engineering design, it is necessary to further determine the appropriate strength reduction factors to obtain the design strength of these systems. Determining appropriate strength reduction factors requires conducting experiments to understand the distribution of the

nominal capacity, which is beyond the scope of this work. Future work can evaluate these distributions. In addition, after scaling the proposed systems to be larger, we expect each connector to have more bolts. In this situation, placement of bolts can also affect the load-carrying capability of connectors.

S7.2 Experiment Details

Here, we show the experiment details. An Optotrack system is used for displacement tracking and a NI DAQ data acquisition tool is used for strain measurement. Strain gauges and optical displacement trackers are attached to the bridge to track local strains and deformations. Before running the experiment, we ran simulation to check the strain distribution and apply strain gauges at those locations with large strains.

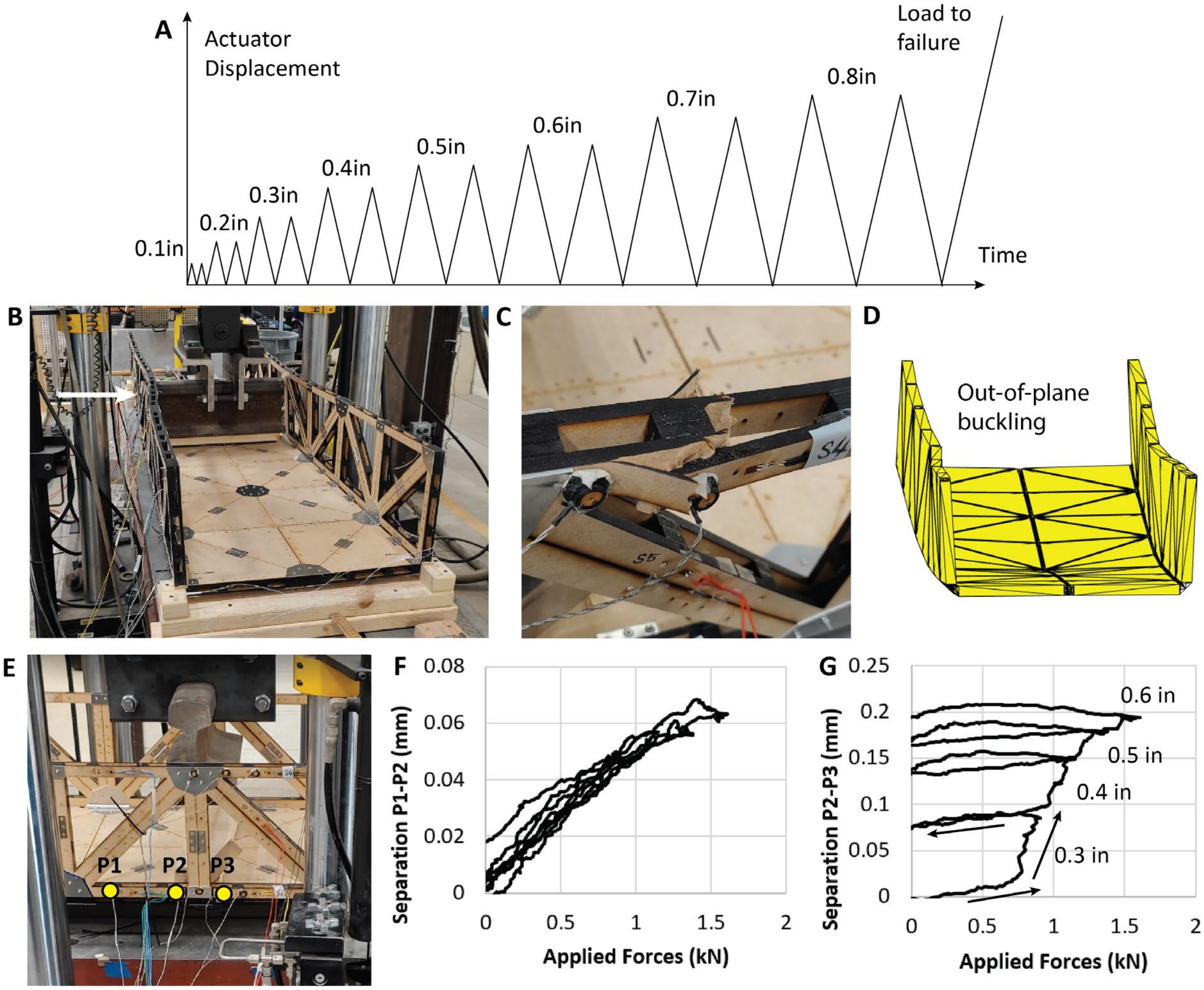

Figure S16. Load carrying experiments of the deployable bridge.

A deformation controlled cyclic loading scheme is used (see Fig. S16A). The structure is incrementally pushed downward at the mid span with increasing deformation each cycle and the applied force and member strains are tracked. The mid-span deflection is increased from 0.1 inch to 0.8 inch with a 0.1 inch increment. Each loading step is repeated twice before increasing the

deformation to the next level. The deformation is controlled using an internal sensor in the Instron Beam Tester. The loading rate is set to be 0.2 inch/minute for both the loading and unloading process. This slow rate ensures that dynamic effects are negligible. When plotting the force-displacement curve shown in Fig. 6 of the manuscript, we use the optically measured displacement at the mid-span of the truss.

After finishing the second 0.8 inch loading-unloading cycle, a final loading step is implemented to push the MUTOIS bridge to failure. Figure S16C and S16D show the failure process. The top truss panels buckle out-of-plane due to compressive internal forces when the structure is loaded to about 1.5 kN (see Fig S16B). At this point, the structure will not fail immediately, and the post-buckling strength can continue to support larger forces. After loading to around 2.6 kN, the MUTOIS bridge reaches its ultimate strength and the truss panel with the largest compressive force (at the mid-span) fails under combined bending and compression (Fig. S16C).

We use data from the optical displacement sensor to show that the hysteresis in the load-displacement curve (in Fig. 7) is due to sliding behaviors from connectors. We monitored separation between sensors P1-P2 (Fig. S16F) and sensors P2-P3 (Fig. S16G) throughout the loading experiment. Sensors P1 and P2 are located at the two ends of a single truss panel with no connectors in between while sensors P2 and P3 are placed at adjacent truss panels that are connected with a gusset plate (see Fig. S16E). Results show that the separation between sensors P1-P2 is mostly linear with limited hysteresis but the separation between sensors P2-P3 has a strong hysteresis due to connectors sliding.

This sliding behavior occurs in the connectors because we use standard bolts. When using standard bolts, there is limited friction between the gusset plate and the connected MDF member. When applying cyclic loading, there will be sliding between the gusset plate and the MDF member, which produces hysteresis. Figure S17 shows how slacks between bolts and connectors can produce this hysteresis. When tension is applied, gaps will form in the connectors until the bolts come into contact with connection plates and MDF; when compression is applied, gaps will be closed and there is direct contact between MDF. In Civil Engineering practice, we can replace these standard bolts with high-strength bolts to prevent sliding behaviors. High-strength bolt can produce a high friction force, which stops the relative motion between the gusset plate and the wood member. Another way to stop the relative motion is to use wood screw rather than the M3 machine screws. However, using wood screw may cause the structure to be no longer reusable.

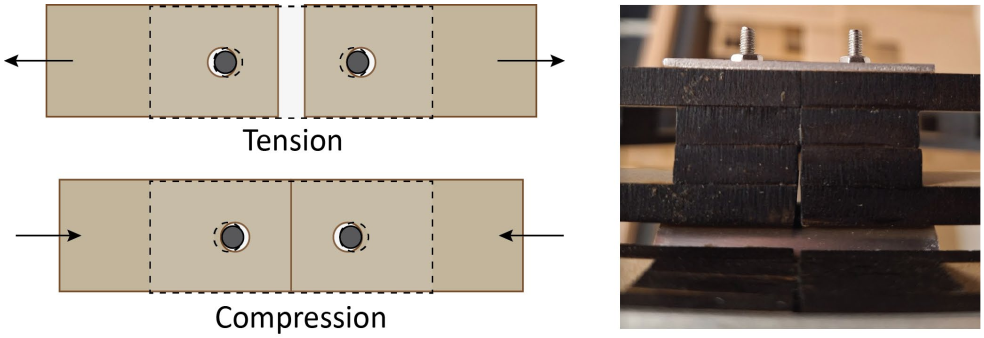

Figure S17. Hysteresis in connectors

S8 Load-Carrying Experiment of the MUTOIS Column

After the MUTOIS bridge (in Fig. S16) is loaded to failure, the undamaged triangle panels are reused and repurposed as a MUTOIS column (process shown in Supplementary Movie 4). Here, we show an axial compressive experiment of this repurposed MUTOIS column. The loading experiment is done using the Instron Beam Tester, where the specimen is placed under the loading head and compressed (see Fig. S18A). Optical deformation sensors and strain gauges are attached to the model for data acquisition. The force output and the actuator deformation are tracked using the sensor within the Instron Beam Tester. The bottom end of the column sees no rotational during the experiment, but the top of the column can rotate. This is because the testing machine uses a structural hinge to connect the actuator and the loading plate (Fig S18A).

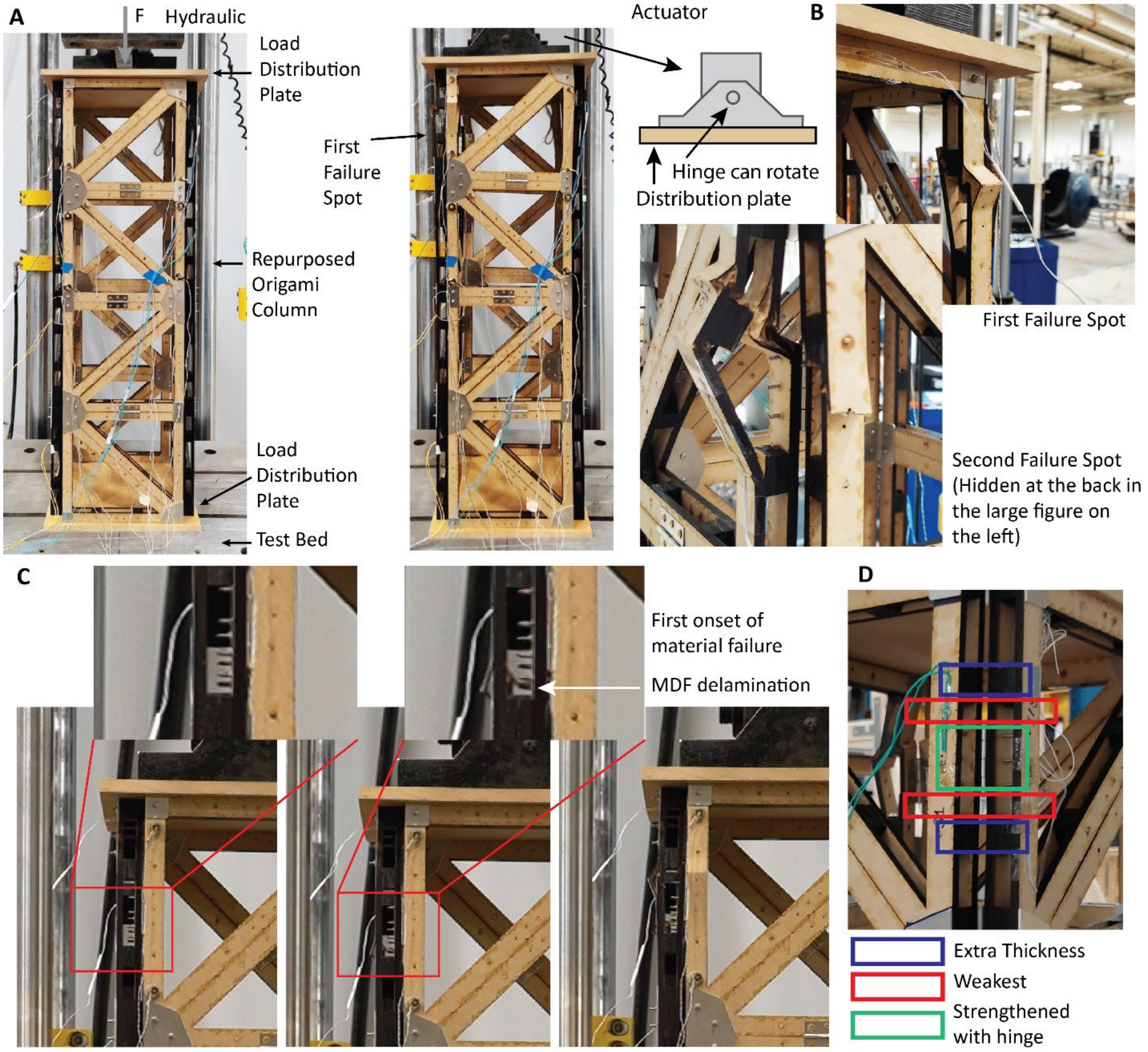

Figure S18. Experimental testing setup for the axial column.

Before loading to failure, the column is first loaded with a small compression cycle to check sensor status (with 0.08 inch or 2 mm deformation). At this axial compression level, the column undertakes about 400 lbs or 1.8 kN of forces. After running this test procedure twice, the column is directly loaded to failure. Before the first failure of the truss member, the column reaches a loading capacity of 21 kN (2.1 tons). After the first truss fails, the system capacity drops, but the column can continue to carry 15 kN (1.5 tons) of force (see Fig. 7).

Strain histories from a selected vertical truss and diagonal truss elements are shown in Fig. S19, and the vertical truss is not the two that failed during the experiment. We see that only the vertical truss develops significant strain during the experiment. This is expected because the diagonal truss members are not contributing to resisting the pure compressive load. From the strain history of the vertical truss member, we can also identify the occurrence of the first and second truss failures.

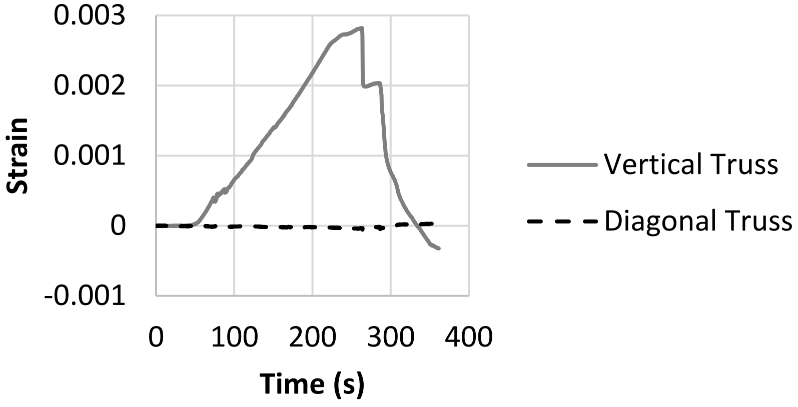

Figure S19. Strain measurement of a vertical and a diagonal truss member during experiment.

We can show that compressive material failure is the major cause of failure. The total cross section area of this column is:

$$A_{col} = 16 \times (0.64cm \times 2cm) = 20.48cm^2$$

Thus, with the known 10MPa compressive strength of MDF, we know that the compressive strength of this column is:

$$F = A_{col} \times \sigma_{max} = 20.48\ kN$$

This theoretical maximum force is close to the capacity obtained in the experiments, showing that compressive material failure is indeed the major cause.

Figure S18C also shows the onset of column failure. The first sign of column failure is delamination of MDF, which is a sign of compressive material failure. Figure S18D further shows that the failure occurs at the weakest point along the height of the vertical struts. Next to this location, there are truss members with added thickness or added metal hinges. Both the additional thickness and the hinge plate can strengthen the cross section.

S9 Mid-Density-Fiber Board (MDF) and Connector Strength Testing

Here, we discuss the material testing for MDF wood panels. We tested five tensile samples to obtain material properties of the MDF wood panels. The tensile test is executed following the ASTM D3500-20 standard. The specimens are cut out using the Universal Laser System laser cutter, and the experiment is done using MTS 810 Material Test System. The displacement is tracked using the Optotrack system. The strain is calculated using optically measured distance between tracking points that are spaced about 2.5 inch (6.3 cm) apart. The forces are measured using force gauge reading of the MTS machine. The material testing setup and the specimens are shown in Fig. S20.

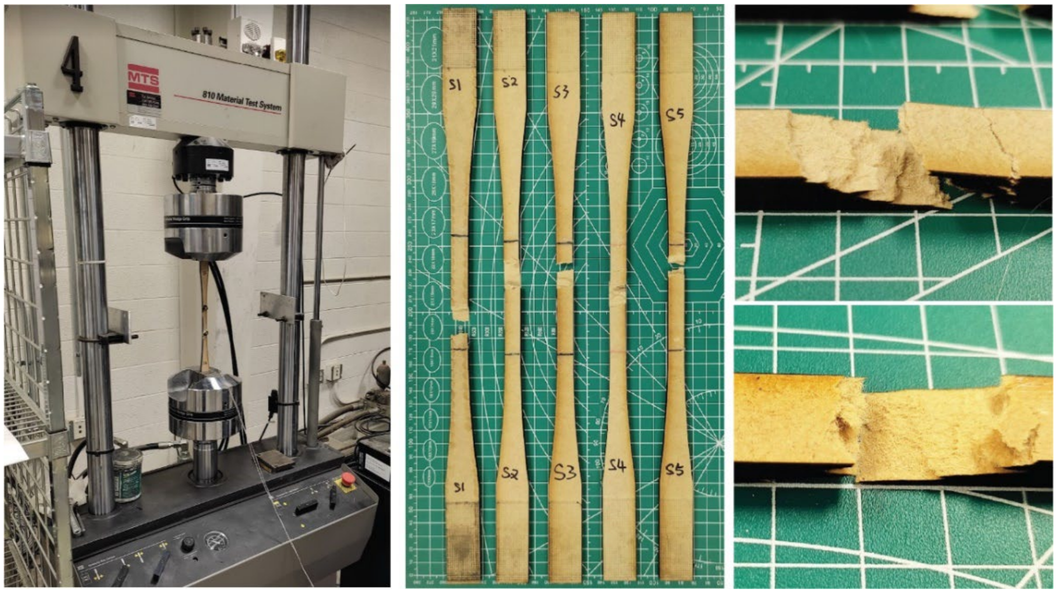

Figure S20. Material testing of MDF board

The stress-strain curve from the material testing is shown in Fig. S21. The MDF board has relatively repeatable material properties, and the variability across five samples is small. The average Young's Modulus of MDF is 3.2 GPa and the average failure stress is 18 MPa. This tensile failure stress is close to the same as the documented 18 MPa tensile strength provided by the manufacturer [32]. Thus, instead of testing the compressive stress, we use 10 MPa as the compressive strength, which is the value provided on the datasheet [32]. There is no obvious yielding point in the material response, which is typical for these wood products. A relatively sudden failure will occur when the material reaches its maximum stress capacity. Right sub figures from Fig. S20 show close-up pictures of delaminated and cracked cross-sections.

This MDF material is not a strong material, so they are not typically used in modern structural engineering applications. However, we selected this material for prototyping MUTOIS because it has uniform and isotropic material properties, which are good for investigating and understanding the behaviors of a new structural system. In practice, using steel or other structural wood products (like CLT) can produce MUTOIS with higher load-carrying capability.

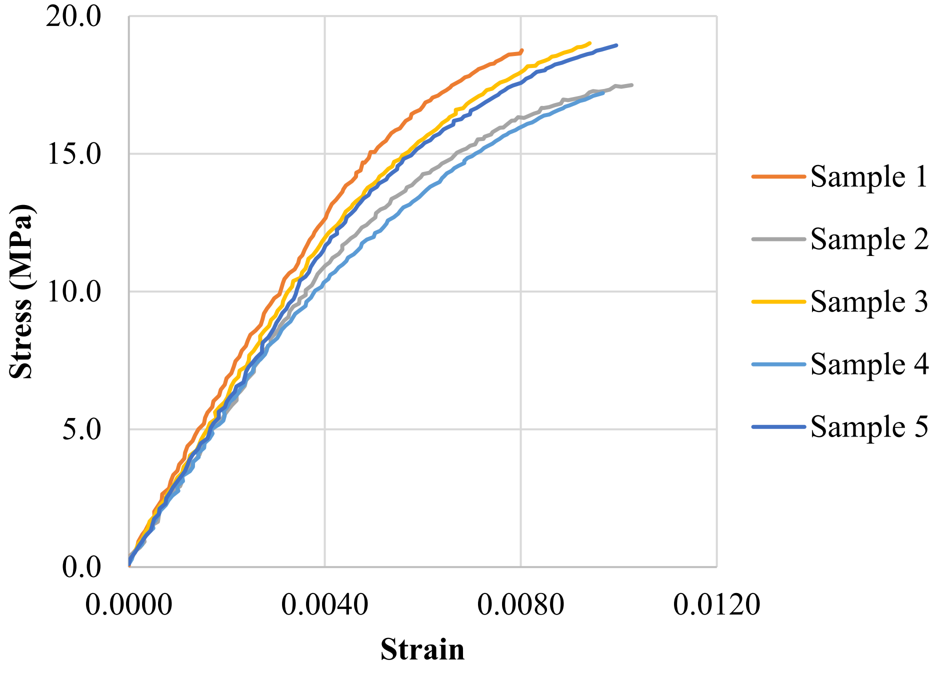

Figure S21. Stress-strain curves of MDF samples tested in tension.

This work also uses the same experiment setup to test the strength and stiffness of the connector plate. More specifically, we want to show that uniform thickness is important for creating origami structures with good load-carrying capability. Figure S22 shows the experiment setup and the tested connector samples. Figure S22A shows a hinge design with non-uniform thickness, where one side of the hinge is 1 inch thick while the other side of the hinge is 0.5 inch thick. To properly lock this hinge, a bent connection gusset plate is necessary. Figure S22B shows another hinge design with uniform thickness, where both sides of the hinge are 0.5 inch thick. This design can be connected using a straight connection plate.

Both designs are tested using the MTS loading machine as illustrated in Fig S22C. The loading rate is set to be 1mm/min according to the ASTM recommendation. This loading rate is slow enough to avoid dynamic effects. The displacement and the applied load are tracked using the internal sensor from the MTS loading machine.

Figure S22D shows the loading results of both samples. The curves shows that the uniform thickness hinge can provide better strength and stiffness when compared to a hinge with non-uniform thickness. The uniform thickness hinge system can achieve 2.7 kN of ultimate load and 5.1 GPa initial stiffness. On the contrary, the non-uniformly thick hinge can only achieve a 1.35 kN ultimate load and 1.1 GPa initial stiffness. This behavior is expected because the bent connector plate cannot offer comparable strength and stiffness when compared to the straight connector plate. The bending deformation in the bent connector plate makes the connector softer when compared to their straight counterpart. This phenomenon is confirmed with the failure

mode displayed in Fig. S22A and Fig. S22B. In the bent connector, the plate is bent as the gap opens up between the two ends. We do not see damage in the Al connector plate near the connector screws, indicating that the internal force within the plate is small. In the straight connector, the connection hole is substantially enlarged by the screws, indicating a large force within the Al plate. In both experiments, the load-displacement behavior are highly ductile – which is an ideal characteristic for civil engineering applications.

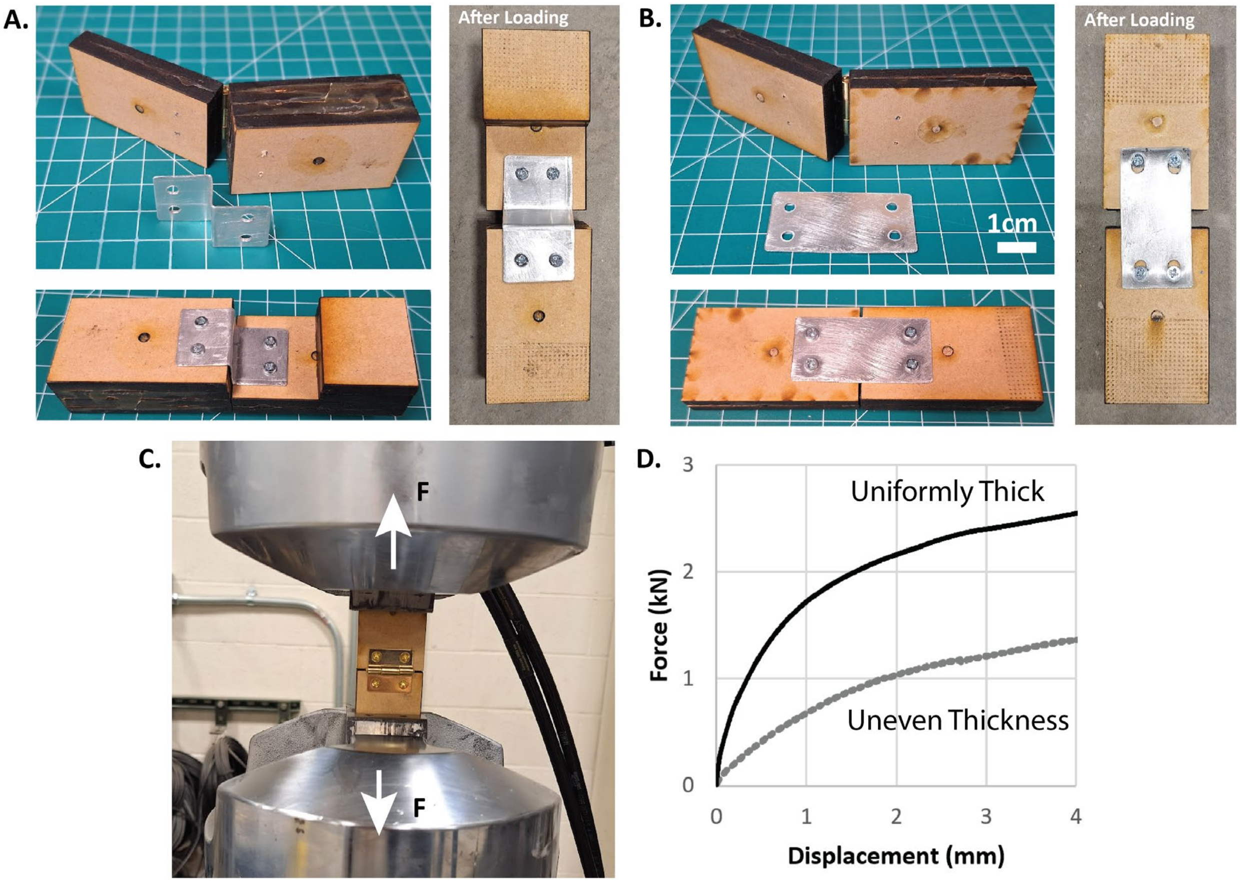

Figure S22. Connector experiment demonstrating higher stiffness and capacity of systems with uniform thickness.

S10 Extrapolating Column Testing Data

Here, we show details regarding how we extrapolate the MUTOIS column testing result to an expected behavior of the same column but made with other common Civil Engineering materials. We assume that the volume and the geometry of the MUTOIS column remain unchanged (only the material is different). Therefore, both the self-weight and the strength of the column can be linearly scaled using the material density and material strength. The material strength, modulus of elasticity, and density values are obtained from journal publications, manufacturer datasheets, or industry standards.

When using this linear scaling, it is necessary to assume that the column failure is dominated by material strength failure and not by a buckling failure. From mechanical analysis, we know that the Euler buckling load is:

$$P_{cr} = \pi^2 \frac{EI}{L^2}$$

Where L is the effective length of column, I is the bending rigidity of the column, and E is the Young's modulus. Thus, if we preserve the geometry of the column, we know that the buckling strength of the column will scale with the Young's Modulus linearly – buckling strength scales with stiffness. Similarly, we know that the column failure strength under material failure can be determined using the equation:

$$P_u = \sigma_u A_{col}$$

Where σ_u is the compressive strength of material and A_{col} is the cross section. If the structure geometry is preserved, changing material will change the σ_u. Therefore, after changing MDF to material A, we know that the column failure strength under material failure and under stability failure can be computed as the following:

$$P_u^A = P_u^{MDF}\left(\frac{\sigma_A}{\sigma_{MDF}}\right)$$

$$P_{cr}^A = P_{cr}^{MDF}\left(\frac{E_A}{E_{MDF}}\right)$$

From our experiment, we can confirm that material failure is the major cause for the MDF column, so we have:

$$P_{cr}^{MDF} > P_u^{MDF} = \sigma_u A_{col} = 20.5kN$$

$$\frac{P_{cr}^{MDF}}{P_u^{MDF}} > 1$$

In this case, because P_u^{MDF} is the smaller force, it controls the failure mode (i.e. we obtain material failure). Then, we can derive a factor F to determine if the MUTOIS column may be affected by a stability failure after switching to material A.

$$\frac{P_{cr}^{A}}{P_{u}^{A}} = F\frac{P_{cr}^{MDF}}{P_{u}^{MDF}} = \frac{P_{cr}^{MDF}\left(\frac{E_A}{E_{MDF}}\right)}{P_{u}^{MDF}\left(\frac{\sigma_A}{\sigma_{MDF}}\right)} = \frac{P_{cr}^{MDF}}{P_{u}^{MDF}}\frac{E_A}{E_{MDF}}\frac{\sigma_{MDF}}{\sigma_A}$$

$$F = \frac{E_A}{E_{MDF}}\frac{\sigma_{MDF}}{\sigma_A}$$

If the F factor is greater than 1, the increase in stiffness is more than the increase in material strength. In this case, we know that the buckling load P_{cr}^{A} is still larger than material failure load P_{u}^{A}. In this situation, material failure should still be the leading cause.

Using this factor F, we can determine if additional consideration is needed for the buckling failure after switching the material from MDF to other building materials. The calculation results are summarized in Table S6. We can see that after switching the material to steel or ultra-high-performance concrete (UHPC), we obtain an F factor that is greater than 1. Thus, we expect material failure to dominate these two cases. However, we see that switching the material to aluminum and cross laminated timber (CLT) can produce an F factor that is smaller than 1. In these situations, additional investigation is needed to evaluate the possibility of a stability failure. In addition, after switching the material, other failure modes can also occur, but these cases will require further investigation and are beyond the scope of this work. Nevertheless, we believe the proposed scaling is enough to show that through changing material and enlarging the cross section of our MUTOIS systems, we can obtain stiffness and strength that are comparable to non-deployable civil structures.

Table S6. Extrapolate Column Testing Data to Civil Materials

**Material**	**Column Strength (kN)**	**Column Weight (kg)**	**Material Strength (MPa)**	**Young's Modulus (GPa)**	**Scaling Factor F**	**Density (kg/m3)**
MDF	21	7.4	10	3.2	1	700
Steel	490	84.6	420 [33]	200 [33]	1.5	8000 [33]
UHPC	116.7	26.4	100 [34]	40 [34]	1.25	2500 [34]
Aluminum (5052)	***266***	28.5	228 [35]	70 [35]	0.96	2700 [35]
CLT	***75.8***	6.9	30 [37]	5 [37]	0.52	650 [36]

References

[1] S. D. Guest and S. Pellegrino, "A new concept for solid surface deployable antennas," *Acta Astronautica,* vol. 38, pp. 103-113, 1996.

[2] C. Guang and Y. Yang, "An Approach to designing deployable mechanisms based on rigid modified origami flashers," *Journal of Mechanical Design,* vol. 140, pp. 082301-1, 2018.

[3] R. J. Lang, S. Magleby and L. Howell, "Single Degree-of-Freedom Rigidly Foldable Cut Origami Flashers," *Journal of Mechanisms and Robotics,* vol. 8, p. 031005, 2016.

[4] D. Bolanos, K. Varela, B. Sargent, M. A. Stephen, L. L. Howell and S. P. Magleby, "Selecting and optimizing origmai flasher pattern configurations for finite-thickness deployable space arrays," *Journal of Mechanical Design,* vol. 145, p. 023301, 2023.

[5] R. Y. E. M. D. A. Les Johnson, "Status of solar sail technology within NASA," *Advances in Space Research,* vol. 48, pp. 1687-1694, 2011.

[6] M. D. Rhode and M. M. Mikulas, "Deployable Controllable Geometry Truss Beam," NASA Technical Memorandum 86366, Hampton Virginia, 1985.

[7] Y. Li, Y. Chen, T. Li, S. Cao and L. Wang, "Hoberman-sphere-inspired lattice metamaterials with tunable negative thermal expansion," *Composite Structures,* vol. 189, pp. 586-597, 2018.

[8] L. Puig, A. Barton and N. Rando, "A review on large deployable structures for astrophysics missions," *Acta Astronautica,* vol. 67, pp. 12-26, 2010.

[9] Y. Li and J. Yin, "Metamorphosis of three-dimensional kirigami-inspired reconfigurable and reprogrammable architected matter," *Materials Today Physics,* vol. 21, p. 100511, 2021.

[10] Y. Li, Q. Zhang, Y. Hong and J. Yin, "3D Transformable Modular Kirigami Based Programmable Metamaterials," *Advanced Functional Materials,* vol. 31, no. 43, p. 202105641, 2021.

[11] S. Liu and Y. Chen, "Myard linkage and its mobile assemblies," *Mechanism and Machine Theory,* vol. 44, pp. 1950-1963, 2009.

[12] K. Yamaguchi, H. Yasuda, K. Tsujikawa, T. Kunimine and J. Yang, "Graph-theoretic estimation of reconfigurability in origami-based metamaterials," *Materials and Design,* vol. 213, p. 110343, 2022.

[13] W. Liu, H. Jiang and Y. Chen, "3D Programmable Metamaterials Based on Reconfigurable Mechanism Modules," *Advanced Functional Materials,* vol. 21, no. 9, p. 202109865, 2021.

[14] A. W. Lacey, W. Chen, H. Hao and K. Bi, "Structural response of modular buildings - An overview," *Journal of Building Engineering,* vol. 16, pp. 45-56, 2018.

[15] S. H. Lawson, "AFCEC conducts CONEX dorm field testing," Accessed in October 2023, "https://www.afcec.af.mil/News/Article-Display/Article/1157092/afcec-conducts-conex-dorm-field-testing/", 2017.

[16] H.-T. Thai, T. Ngo and B. Uy, "A review on modular construction for high-rise buildings," *Structures,* vol. 28, pp. 1265-1290, 2020.

[17] J. Brutting, J. Desruelle, G. Senatore and C. Fivet, "Design of Truss Structures Through Reuse," *Structures,* vol. 18, pp. 128-137, 2019.

[18] A. P. Thrall and C. P. Quaglia, "Accordion shelters: A historical review of origami-like

deployable shelters developed by the US military," *Engineering Structures,* vol. 59, pp. 686-692, 2014.

[19] L. Vector, "Inflatable Structures," https://www.vector-build.com/eng_pnevmo.htm, Accssed in 2023.

[20] A. Michler, "Rolling Bridge," eVolo, "https://www.evolo.us/rolling-bridge-thomas-heatherwick/", Accessed in Oct 2023, 2011.

[21] G. R. Thomas and B. J. Sia, "A Rapidly Deployable Bridge System," in *Structural Congress 2013*, 2013.

[22] B. R. Russell and A. P. Thrall, "Portable and Rapidly Deployable Bridges: Historical Perspective and Recent Technology Developments," *Journal of Bridge Engineering,* vol. 18, pp. 1074-1085, 2013.

[23] Military-Today, "M60 AVLB," "https://www.militarytoday.com/engineering/m60_avlb.htm", Accesssed in 2023, 2023.

[24] N. A. Megahed, "An exploration of the control strategies for responsive umbrella-like structures," *Indoor and Built Environment,* vol. 27, pp. 7-18, 2018.

[25] T. G. Berry, "Erectable Structures," US patent US3606719A, 1968.

[26] Y. Chen, R. Peng and Z. You, "Origami of thick panels," *Science,* vol. 349, pp. 396-400, 2015.

[27] T. Tomohiro, "Simulation of Rigid Origami," *Origami 5, CRC Press, ,* pp. 253-264, 2009.

[28] E. T. Filipov, K. Liu, T. Tachi, M. Schenk and G. H. Paulino, "Bar and hinge models for scalable analysis of origami," *International Journal of Solids and Structures,* vol. 124, pp. 26-45, 2017.

[29] M. Schenk and S. D. Guest, "Origami Folding: A Structural Engineering Approach," in *Origami 5*, (July 13-17, 2010, Singapore, CRC press, 2010, 2010.

[30] matweb.com, "Aluminum 6061-T6; 6061-T651," https://asm.matweb.com/search/SpecificMaterial.asp?bassnum=ma6061t6, accessed 2023.

[31] E. Edge, "Loading Capacities BS449," https://www.engineersedge.com/hardware/loading_capacities_bs449_bolt_grade_129__148 22.htm, accessed 2023.

[32] MakeltFrom.com, "(MDF), Medium Density Fiberboard," MakeltFrom.com, https://www.makeitfrom.com/material-properties/Medium-Density-Fiberboard-MDF, accessed 2023.

[33] "European structural steel standard EN 10025: 2004".

[34] M. A. Bajaber and I. Y. Hakeem, "UHPC evolution, development, and utilization in construction: a review," *Journal of Materials Research and Technology,* vol. 10, pp. 1058-1074, 2021.

[35] MatWeb, "Aluminum 5052," in *https://www.matweb.com/*, 2023.

[36] Z. Tian, Y. Gong, J. Xu, M. Li, Z. Wang and H. Ren, "Predicting the Average Compression Strength of CLT by Using the Average Density or Compressive Strength of Lamina," *Forests ,* vol. 13, no. 4, p. 591, 2022.
