# Supplementary material for: Large-scale modular and uniformly thick origami-inspired adaptable and load-carrying structures
Source: Nat Commun. 2024 Mar 15;15:2353. doi: 10.1038/s41467-024-46667-0 (PMC10942996; doi:10.1038/s41467-024-46667-0)
Supplement: Supplementary file 3 — Description of Additional Supplementary Files [file 41467_2024_46667_MOESM3_ESM.pdf]

Description of Additional Supplementary Files

File Name: Supplementary Data 1
Description: Supplementary Laser Cutter File

File Name: Supplementary Movie 1
Description: MUTOIS for Adaptive Structures

File Name: Supplementary Movie 2
Description: Demonstration of Locking Devices

File Name: Supplementary Movie 3
Description: Reusable MUTOIS Bridge

File Name: Supplementary Movie 4
Description: Repurpose MUTOIS Bridge after Damage

File Name: Supplementary Movie 5
Description: Navigate Multi-Path Folding

File Name: Supplementary Movie 6
Description: MUTOIS Bridge Comparison

File Name: Supplementary Movie 7
Description: MUTOIS Bridge Loading Experiment

File Name: Supplementary Movie 8
Description: MUTOIS Column Loading Experiment

File Name: Supplementary Code 1
Description: Simulation for MUTOIS
