# Supplementary figures and images for: Large-scale modular and uniformly thick origami-inspired adaptable and load-carrying structures
Source: Nat Commun. 2024 Mar 15;15:2353. doi: 10.1038/s41467-024-46667-0 (PMC10942996; doi:10.1038/s41467-024-46667-0)

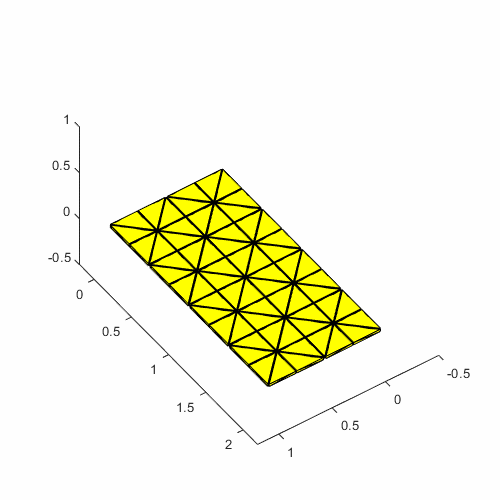

Supplement: Supplementary file 13 — Supplementary Code 1 [file 41467_2024_46667_MOESM13_ESM.zip › SuppCode/MUTO_FullBridge.gif]

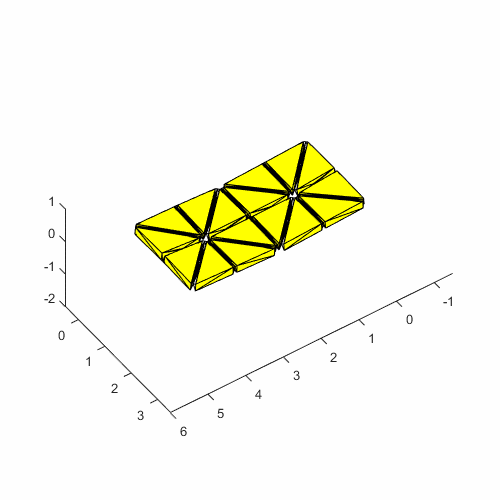

Supplement: Supplementary file 13 — Supplementary Code 1 [file 41467_2024_46667_MOESM13_ESM.zip › SuppCode/MUTO_Section_CFold.gif]

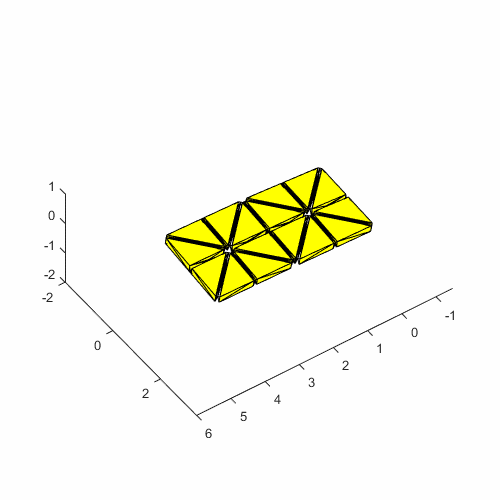

Supplement: Supplementary file 13 — Supplementary Code 1 [file 41467_2024_46667_MOESM13_ESM.zip › SuppCode/MUTO_Section_SkewedCFold.gif]

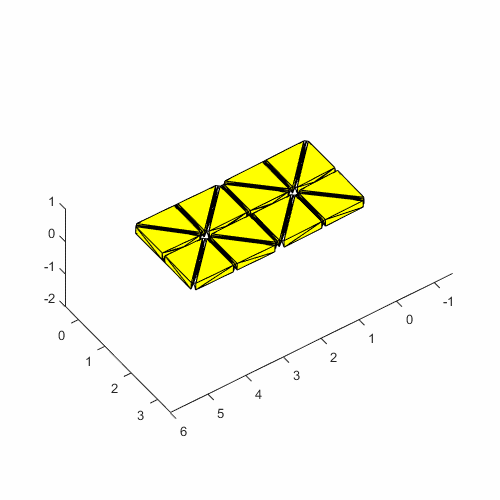

Supplement: Supplementary file 13 — Supplementary Code 1 [file 41467_2024_46667_MOESM13_ESM.zip › SuppCode/MUTO_Section_YoshimuraFold.gif]
